# RNase Y mediates posttranscriptional control of the virulence-associated CncR1 small-RNA in *Helicobacter pylori*

**DOI:** 10.1016/j.isci.2025.111815

**Published:** 2025-01-16

**Authors:** Federico D’Agostino, Eva Pinatel, Alexandra Meynhardt, Vincenzo Scarlato, Andrea Vannini, Davide Roncarati

**Affiliations:** 1 Department of Pharmacy and Biotechnology, Alma Mater Studiorum – University of Bologna, Bologna, Italy; 2 Institute of Biomedical Technologies - National Research Council, Segrate, Italy

**Keywords:** Human metabolism, Cell biology

## Abstract

Ribonucleases are involved in several biological processes, including the turnover of structural and messenger RNAs and the specific processing of the cellular transcriptome. Here, we characterized the RNase Y from *Helicobacter pylori*. We found that RNase Y is membrane-associated and its expression is controlled during bacterial growth and by Fur in response to iron. We observed that RNase Y deletion has a limited impact on *H. pylori* transcriptome and on bacterial growth. Interestingly, we found that RNase Y is involved in the metabolism of CncR1, a virulence-associated sRNA oppositely modulating bacterial motility and adhesion to host cells. Indeed, RNase Y inactivation led to the accumulation of a 3′-extended CncR1 isoform, which appeared unable to interact *in vitro* with a known target mRNA. The observation that the RNAse Y-mutant strain showed deregulation of several members of the CncR1 regulon suggests this ribonuclease has an important role in *H. pylori* posttranscriptional regulation.

## Highlights

• RNase Y in *H. pylori* is membrane associated and regulated by growth and iron
• RNase Y is non-essential, but its deletion affects expression of several virulence factors
• RNase Y mediates the processing of CncR1, a virulence-associated sRNA of *H. pylori*
• Inactivation of RNase Y impacts CncR1 regulon, highlighting its regulatory role

## Graphical abstract

**Figure:**
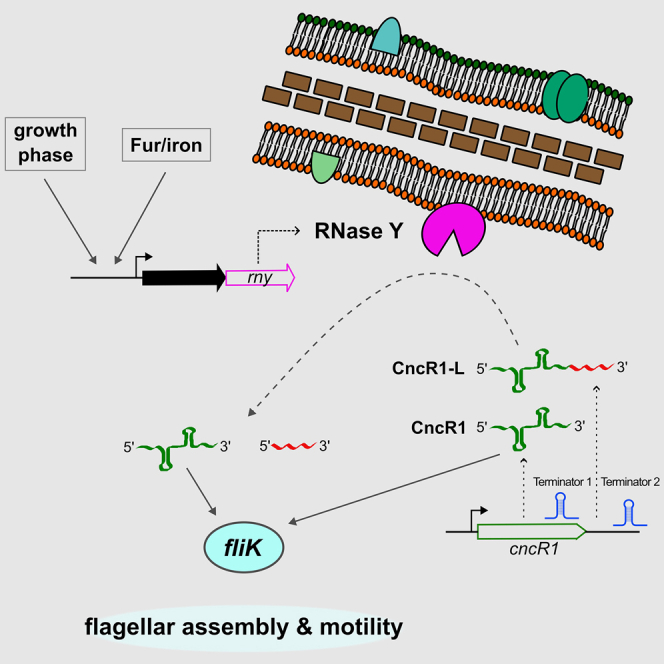

## Introduction

The control over RNA synthesis and decay is a strictly regulated process that allows an adaptive response of bacterial cells to dynamic environmental conditions. Endo- and exo-ribonucleases (RNases) serve as nucleolytic agents involved in the degradation, stabilization, or maturation of different RNA classes (i.e., mRNAs, tRNAs, rRNAs, and ncRNAs). The main RNases that participate in the initial step of RNA metabolism in gram-negative and gram-positive bacteria are RNase E and RNase Y, respectively.^1^ Although RNases act as critical factors in post-transcriptional regulation processes, these ubiquitous enzymes show a complex heterogeneity in composition, number and function across different bacterial species. For instance, except for toxins with ribonucleolytic activity, *Escherichia coli* harbors 16 different RNases, namely 7 exo-RNAses and 9 endo-RNAses. The latter group includes the essential RNase E, which is known to be involved in mRNA turnover,^2^ 16S and 23S rRNAs processing and degradation,^3^ sRNA cleavage,^4^ and maturation of tRNAs and tmRNA.^5^ On the other hand, the gram-positive *Bacillus subtilis* lacks an RNase E ortholog among the 20 different RNases represented in its genome but retains both the functionally related RNase J1/J2 and RNase Y, considered as the functional equivalent of *E. coli* RNase E.^6^^,^^7^

*Helicobacter pylori* is a gram-negative, microaerophilic pathogen which exclusively colonizes the human stomach of more than 50% of the human population, with higher prevalence in Africa (79.1%), Latin America (63.4%), and Asia (54.7%).^8^ When untreated, *H. pylori* infections have been related to many gastrointestinal disorders, including chronic and active gastritis,^9^ gastric and duodenal ulcer diseases,^10^ MALT (mucosa-associated lymphoid tissue) lymphoma, and gastric adenocarcinoma.^11^^,^^12^ Given the low redundancy degree of its small genome (1.6 Mb),^13^^,^^14^ *H. pylori* is equipped with a remarkably constrained number of just 8 predicted RNases: RNase J (HPG27_RS07070), RNase Y (HPG27_RS03700), RNase R (HPG27_RS06255), RNase III (HPG27_RS03230), RNase P (HPG27_RS07175), PNPase (HPG27_RS06080), RNase HI (HPG27_RS03225), and RNase HII (HPG27_RS06655). The essential 5′ exo- and endo-RNase J plays a fundamental role in *H. pylori* physiology. Indeed, RNase J constitutes the minimal *H. pylori* degradosomal complex together with the DExD-box helicase DeaD (RhpA in *H. pylori* 26695 strain).^15^^,^^16^ These two proteins are compartmentalized into foci located at the inner cell membrane and are associated with translating ribosomes.^17^ Depletion of RNase J levels in *H. pylori* B128 strain was associated with a 4-fold increase of 55% of the mRNAs and 49% of the asRNAs. However, the transcript levels of only 5 sRNAs encoded from intergenic regions (IGRs) were increased more than 4-fold, and no role in the maturation of other stable ncRNAs (i.e., tRNAs and rRNAs) was observed.^18^ Accordingly, the double-strand specific RNase III starts the rRNA processing by cleaving two stem-loop structures in the 23S-5S rRNA polycistronic precursor and a stem-loop upstream of the mature 5S sequence. Also, RNase III cleaves the intermolecular complex formed by the leader region of the 23S-5S precursor and its *cis*-encoded asRNA.^19^ Few studies shed light on other *H. pylori* ribonucleases. The 3′-5′ exo-RNase R was found to form complexes anchored to the inner cell membrane with RhpA helicase, but its deletion has only a minor impact on the global RNA decay.^20^

The RNase Y is the only other single-strand specific endo-RNase represented in the *H. pylori* genome shown to be central in the degrading machinery of pathogens, such as *B. subtilis*, *Streptococcus pyogenes*, and other low-GC gram-positive bacteria.^21^^,^^22^ The merging of in silico analysis and experimental validation of the domain structure of RNase Y in *B. subtilis* revealed the presence of four major modules. The extended N-terminal α-helical region comprises a short transmembrane domain and an intrinsically disordered coiled-coil domain involved in the subcellular localization of the protein and its oligomerization.^23^^,^^24^^,^^25^ The globular C-terminal region instead harbors a type 1 K-homology domain (KH domain), responsible for target RNA sequence recognition and binding,^26^ and a catalytic HD domain.^27^ RNase Y is thought to have different roles depending on the bacterial species. Deletion of the RNase Y encoding gene (*rny*) in *B. subtilis* led to a significant decrease in growth rate, a 2-fold increase in the bulk mRNA half-life, and more pleiotropic effects on sporulation, competence, antibiotic susceptibility, and cell morphology.^6^^,^^28^ In addition, RNase Y is involved in the maturation of different RNA classes, such as riboswitches,^29^ the ribozyme RNase P,^30^ and polycistronic mRNAs.^31^ A strong impact on global gene expression and on regulating important virulence factors was also revealed for *Clostridium perfringens*.^32^ Nevertheless, such solid activity was not reported for *Staphylococcus aureus* and *S. pyogenes*, where only a limited but exclusive number of direct targets has been identified.^33^^,^^34^ For example, in *S. aureus* the RNase Y cleaves the *saePQRS* operon stabilizing the fragment encoding the SaeRS two-component system.^35^

With this work, we investigate the global role of RNase Y in *H. pylori* for the first time. We adopted an RNA-seq approach comparing a *rny* knock-out mutant to the parental wild-type strain to find out the post-transcriptional effect exerted by the RNase Y. We discovered that RNase Y has an auxiliary role compared to the RNase J, and it is responsible for the regulation of a narrow spectrum of targets. In addition, we find that RNase Y is involved in the metabolism of *cag*-non-coding RNA 1 (*cncR1*), a *trans*-acting sRNA involved in the programmed expression of motility and adhesion genes. Indeed, the absence of a functional RNase Y enzyme led to the accumulation of a 3′-extended CncR1 isoform (CncR1-L), which is unable to bind *in vitro* a known CncR1 target (*fliK*), and so, presumably, to exert a post-transcriptional regulation on it.

## Results

### The RNase Y encoding gene belongs to an operon finely tuned during bacterial growth and in response to iron levels

**Figure 1:**
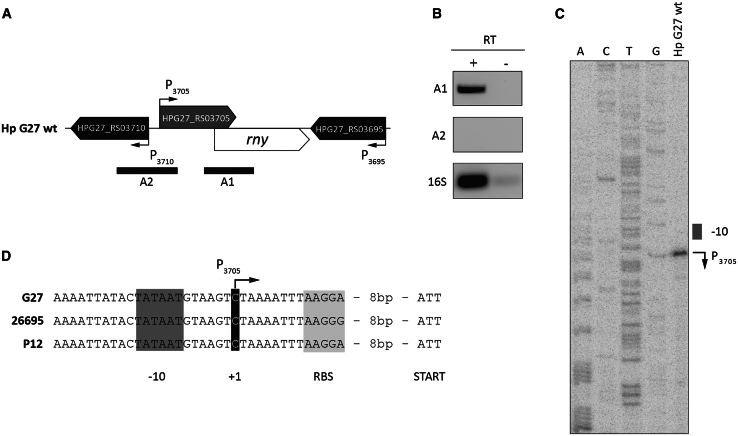
*Helicobacter pylori rny* genetic locus: promoter, transcriptional start site, and operon structure (A) Genomic organization of the *HPG27_RS03705*-*rny* operon in *H. pylori* G27 strain with bent arrows indicating the transcription start sites (TSSs). The position of the predicted amplicons expected by the reverse transcription-PCR (RT-PCR) analyses is indicated by black bars. (B) Agarose gel electrophoresis showing the indicated RT-PCR products. The A1 product overlaps the HPG27_RS03705 and *rny* regions (predicted as depicted in A), generated with oligos 716-F and 717-R. The predicted A2 product (A), encompassing *HPG27_RS03705* and the divergently oriented *HPG27_RS03710* was checked by using oligos 717-F and 718-R resulted in no amplification. A positive control generated on the 16S transcript (amplified with oligos 16S-RTF and 16S-RTR) was included. (C) Primer extension analysis performed on RNA extracted from *H. pylori* G27 strain. Primer extension and Sanger sequencing reactions were carried out using oligo 717pe9. The TSS is indicated by a bent arrow and marked P_3705_. (D) Nucleotide sequence and relevant features of the P_3705_ promoter: −10 box, TSS, and Shine-Dalgarno sequence are embed in dark gray, black, and light gray boxes, respectively. Symbols are as in (C).

In the *H. pylori* G27 strain,^36^ the genomic arrangement of *HPG27_RS03705* and *rny* (*HPG27_RS03700*) genes closely mirrors that observed in the 26695 strain,^37^ with both coding sequences (CDSs) positioned in a tandem orientation and exhibiting an overlap of 101 base pairs (bp) (Figure 1A). To confirm whether the *HPG27_RS03705* and *rny* genes in the G27 strain are transcribed as a single transcriptional unit, we set up reverse transcription-PCR (RT-PCR) on cDNAs derived from a total RNA extracted from *H. pylori* G27 wild-type cells (key resources table). Two genomic regions were assayed: the first region (A1, Figure 1A) encompassed portions of the *HPG27_RS03705* and *rny* CDSs, including their overlapping sequences, while the second region (A2, Figure 1A), serving as a negative control, spanned from *HPG27_RS03705* to the divergently oriented *HPG27_RS03710* gene. Results presented in Figure 1B shows a strong signal corresponding to the A1 amplicon and a complete absence of the A2 amplicon, suggesting that *HPG27_RS03705* and *rny* are transcribed as a single bicistronic unit. In addition, a Sanger sequencing reaction and a primer extension performed with a probe located on the 5′-UTR of *HPG27_RS03705-rny* operon allowed the identification of the TSS, which maps 22 bp upstream of the annotated start codon of the *HPG27_RS03705* CDS (Figure 1C).

**Figure 2:**
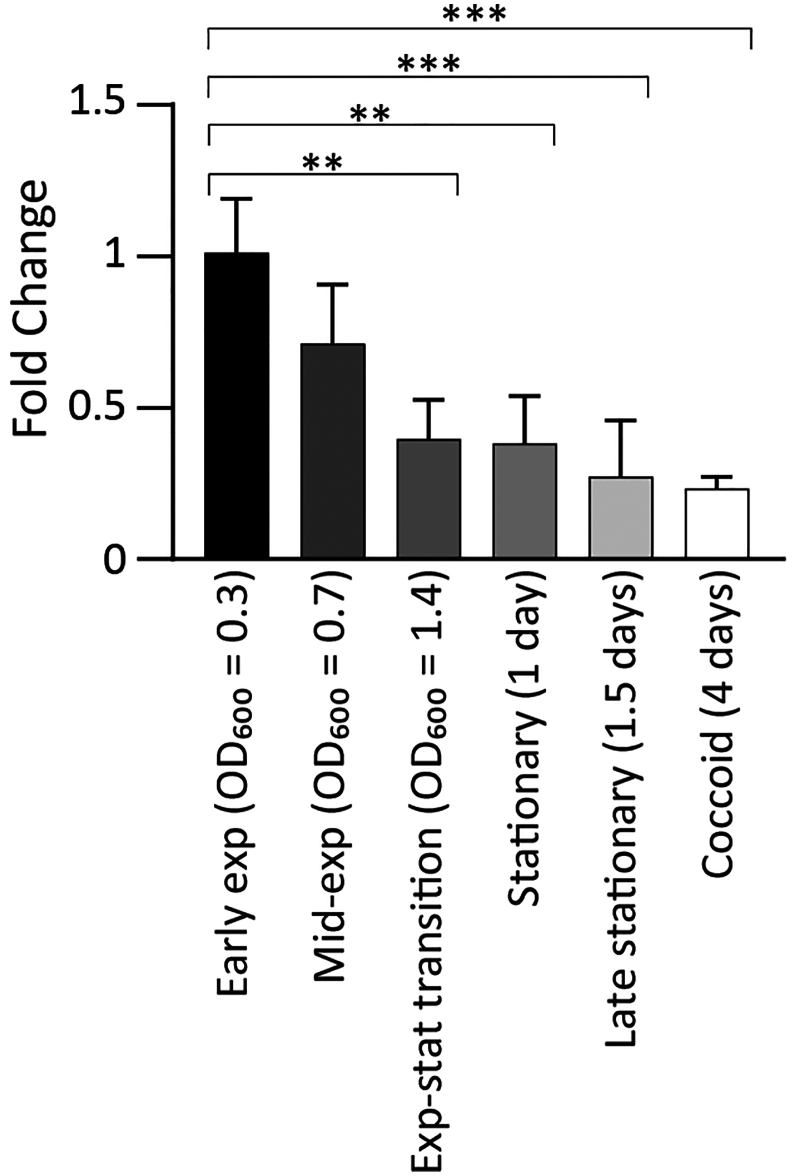
*rny* transcript levels are regulated during bacterial growth Quantitative PCR (RT-qPCR) analysis was performed with oligonucleotides specific for 16S (16S-RTF/16S-RTR) and *rny* (RNaseY-RTF/RNaseY-RTR). Ct values were normalized on the 16S internal control and gene expression is reported as n-fold changes with respect to the early exponential phase of growth. Mean values from 3 biological replicates are reported +/− SD. The statistical significance was calculated with one-way ANOVA test (multiple comparisons vs. “Early exp” condition) and expressed as: ∗∗ = *p* value < 0.01; ∗∗∗ = *p* value < 0.001.

P_3705_ promoter was further characterized. The DNA sequence revealed the presence of a TATAAT -10 box matching the canonical σ^80^ consensus sequence (Figure 1D). Transcript levels of the *HPG27_RS03705*-*rny* operon were previously shown to increase under iron-limiting conditions through indirect positive regulation by HpFur.^38^ Transcriptional analysis of the *HPG27_RS03705*-*rny* operon during bacterial growth showed elevated levels in the early exponential phase, followed by a significant decline in the subsequent growth phases, with lowest levels in the late stationary and coccoid phases (Figure 2).

### Bioinformatics analysis of the *H. pylori* RNase Y structure

The *H. pylori* G27 Rny protein consists of 529 amino acids and is preceded by a ribosome binding site (RBS) (Figure S1A). Notably, some annotations of the *rny* gene in *H. pylori* G27 have erroneously identified a downstream start codon, resulting in a shorter polypeptide of 503 amino acids. Analysis of the DNA sequence in the locus revealed the absence of an upstream RBS (Figure S1A), suggesting that translation of the Rny protein initiates from the upstream start codon. A BLAST-P analysis within *H. pylori* species retrieved thousands of orthologues (database query conducted on 29 October 2024 by BLAST-P with BLOSUM62, word size 5, gap costs −11/-1; identity ≥93% and coverage ≥66%) in other strains (see Table S2; Figure S1B), including the well-characterized 26695, P12, J99, SS1, and HPAG-1 *H. pylori* strains. Accordingly, most of these orthologues correspond to the longer annotated form (i.e., 529 aa long). Similarly, BLAST-P analysis in other non-*H. pylori* species retrieved 98 orthologues (identity ≥50% and coverage ≥60%) in other Helicobacter species (*H. felis*, *H. suis*, *H. bizzozeronii*, *H. cetorum*, and *H. heilmannii*) and related bacteria, as *Campylobacter jejuni*, *Escherichia coli*, and Sulfurovum sp. (see Table S3; Figure S1B). While the C-terminal region shows a high degree of amino acid conservation, the N-terminal region appears to be specific to *H. pylori*.

**Figure 3:**
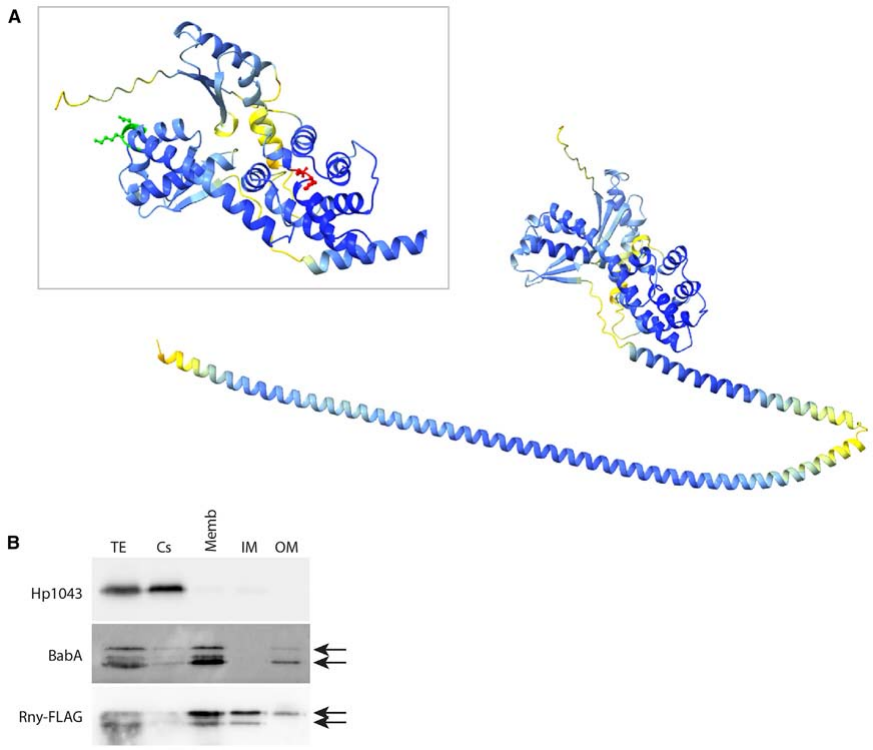
Three-dimensional model of *H. pylori* RNase Y and its localization in the inner membrane (A) The model of the *H. pylori* RNase Y has been obtained using the AlphaFold tool. Inset on the α/β fold globular domain, harboring the predicted KH RNA-binding domain (residues 233–236 in green) and predicted HD catalytic domain (residues 374–375 in red). (B) Western blot analysis of the 5 cellular fractions obtained from the Δ*rny*::*rny*-FLAG *H. pylori* strain by differential solubilization and ultracentrifugation. TE (total extract), Cs (cytosolic soluble fraction), Memb (total membrane fraction), IM (inner membrane fraction), and OM (outer membrane fraction). Antibody against FLAG tag was employed to identify Rny-FLAG (2 specific bands), and antibodies against the cytosolic Hp1043 and OM BabA (2 prominent bands) proteins were used to validate bacterial fractionation. All samples correspond to the same initial number of bacteria. Experiments were performed in triplicate and representative blots are reported.

To better characterize the *H. pylori* RNase Y, we submitted its 529 amino acid sequence to AlphaFold and RoseTTAFold prediction tools, due to their ability to accurately predict protein structures.^39^^,^^40^^,^^41^ According to the similar three-dimensional models generated by the two software (Figures 3A, S2, and S3), the protein is characterized by an N-terminal extended α-helical domain (residues 1–207), made by two long α helices connected by a short loop, and an α/β fold globular domain (residues 216–518) in the carboxyl-terminal part of the polypeptide.

We then used the Dali server to search for similar three-dimensional structures in the Protein Data Bank.^42^ From this analysis, it turned out that the AlphaFold model of the putative RNase Y is similar to structures of proteins possessing RNA-binding capabilities as well as ribonuclease activity. In detail, the top ten Dali server hits include four polynucleotide phosphorylases and six RNA-binding proteins involved in different cellular functions (Table S4). These indications were confirmed and refined by carrying out a functional analysis of the protein with InterPro, which classifies a query protein of interest into families and predicts domains and important sites.^43^ This analysis suggested the presence of a central KH (residues 225 to 297) and HD (residues 342 to 415) domains (Figure 3). The KH domain is a single-stranded, sequence-specific RNA binding domain characterized by a minimal β-α-α-β core structure. The ability to bind the nucleic acid backbone lies in a conserved G-X-X-G motif (identified in the residues 233–236, Figure 3) that links the two α helices of the minimal core.^26^ The HD domain instead was predicted to be the catalytic domain. Considering the high conservation degree in the Helicobacter genus, we identified the His-Asp couplet in position 374–375 (Figure 3) as the Zn^2+^ and Mg^2+^ metal-chelating residues that could coordinate the RNase Y phosphohydrolytic activity.^27^

Analysis of the RNase Y sequence by TMHMM - 2.0 tool (Figure S4A) suggested the presence of an N-terminal transmembrane domain (TM, residues 5 to 27 of the polypeptide of 529 amino acids), with clustered hydrophobic residues (15/23) in the predicted TM region. The importance of the transmembrane domain, which is predicted to cross the bacterial membrane with a single α-helical motif, is further supported by the membrane localization of the RNase Y orthologues in other bacteria, such as *Bacillus subtilis*.^24^ To confirm the localization of the Hp RNase Y in the cell membrane, the strain expressing a C-terminal FLAG-tagged variant of the Rny protein was fractionated into different subcellular fractions and their protein content was assayed by western blot. The proper separation of the different fractions was validated by western blot analysis of the cytosolic soluble (Cs) protein HP1043 and of the BabA adhesin, known to be localized in the outer membrane (OM) (Figure 3B). Determination of the lipopolysaccharide (LPS) content in the different cell fractions confirmed the proper separation of the Cs, inner membrane (IM), and OM fractions (Figure S4B). Western blotting with anti-FLAG antibody showed that Rny is located almost exclusively in the membrane fraction (Memb), as predicted by the bioinformatic analysis. Furthermore, Rny-FLAG is mainly present in the IM fraction (Figure 3B), which is almost uncontaminated by Cs and OM fractions (see Hp1043 and BabA blots, as well as by the LPS staining). A small amount of Rny-FLAG is also detected in the OM fraction, although this signal may result from slight contamination of this fraction by IM (see BabA blot).

The model of *H. pylori* RNase Y protein shows a very long N-terminal alpha-helix (amino acid 3 to 153), including the TM region. Analysis of this sequence with various bioinformatic tools^44^^,^^45^^,^^46^ predicted a coiled-coil region (amino acid 75 to 135) that likely mediates homo- or hetero-multimerization (Figures S5A–S5D).

### RNase Y is a non-essential enzyme involved in the regulation of a narrow spectrum of targets

**Figure 4:**
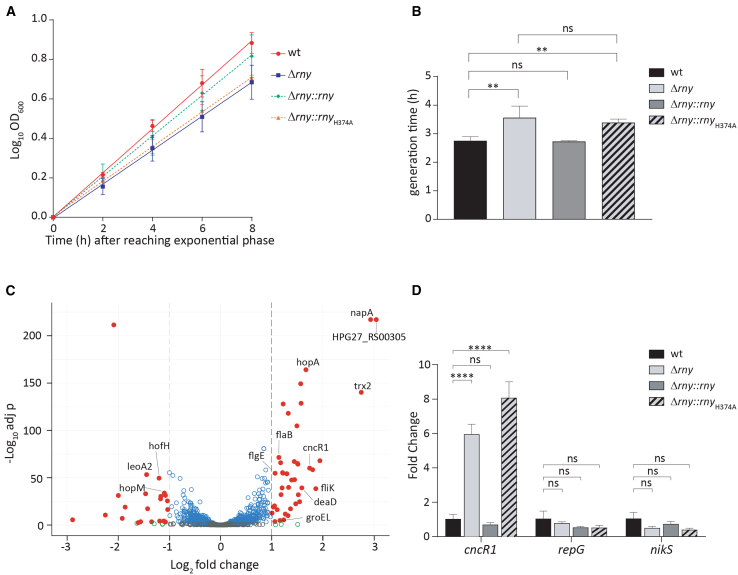
Effects of HP *rny* deletion on bacterial growth, transcriptome, and expression of the noncoding RNA CncR1 (A and B) Fitness assessment of wild type, Δ*rny*, Δ*rny*::*rny*, and Δ*rny*::*rny*_H374A_ strains by measurements of optical density at 600 nm (OD_600_) during exponential growth phase. Log_10_(OD_600_) is plotted against the time after reaching the exponential phase and linear regression was calculated (A). The generation time of each strain was calculated by the following formula: generation time = [duration x ln_2_]/[ln(ODf/ODi)], where ODf and ODi indicate the initial and final optical densities, respectively. (C) Volcano plot of RNA-sequencing (RNA-seq) analysis results. Each point corresponds to the difference in gene expression (Log_2_FC; X axis) between Δ*rny* and wild-type strains (X axis) plotted against its statistical significance (-Log_10_*adj p*; Y axis). Differentially expressed genes (DEGs) are represented with red filled circles when log2FC > |1|, *adj p* < 0.01; empty gray circles correspond to non-differentially expressed genes; empty blue or green circles correspond to genes matching only *adj p* < 0.01 or log_2_FC > |1| criteria respectively. Black dashed lines indicate log_2_FC < 1, log_2_FC > 1 and *adj p* < 0.01 threshold values. (D) Transcript levels of *cncR1*, *repG*, and *nikS* determined by RT-qPCR in Δ*rny* (light gray bars), Δ*rny*::*rny* (dark gray bars), and Δ*rny*::*rny*_H374A_ (striped gray bars) mutant strains compared to wild-type (black bars) strain. Oligonucleotides pairs 536-RTF/536-RTR (CncR1), RepG-RTF/RepG-RTR (RepG), and NikS-RTF/NikS-RTR (NikS) were used. Ct values were normalized on the 16S internal control and gene expression is reported as n-fold variation to the wild-type strain. In (A), (B), and (D) mean values from three biological replicates are reported, ±SEM; in (B) and (D), statistical significance was calculated by one-way ANOVA test (all comparisons for B, multiple comparisons vs. wt for D) and expressed as: ns = not significative; ∗∗ = *p* value < 0.01; ∗∗∗∗ = *p* value < 0.0001.

To investigate on the functional role exerted by RNase Y in *H. pylori*, an isogenic *rny* knock-out mutant (Δ*rny*) was generated. To this purpose, *H. pylori* G27 was transformed with the PCR product rny-UP::KmR:rny-DOWN to replace most of the *rny* CDS with the kanamycin resistance gene cassette,^47^ leaving the upstream *HPG27_RS03705* gene intact. The correct insertion of the Km^R^ gene was assessed by PCR and sequencing. To assess the phenotypic effects exerted by the deletion of *rny* gene, we determined the growth rate during the exponential phase by measuring optical density at 600 nm (OD_600_). This analysis indicated that the deletion of the *rny* was associated with a growth defect of the *rny*-mutant compared to the wild-type strain. Indeed, the generation time significantly increased (∼30%) from 2.75 ± 0.15 h (wt) to 3.57 ± 0.41 h (Δ*rny*), suggesting that the absence of RNase Y has a modest impact on *H. pylori* fitness (Figures 4A and 4B). A similar phenotype was observed in the *H. pylori* P12 *rny* KO-mutant (Figure S6A).

To examine the contribution of the RNase Y in the post-transcriptional degradation processes of different RNA classes, we performed a strand-specific transcriptome analysis of *H. pylori* G27 wild type and Δ*rny* strains. Both strains were grown in the same conditions and bacterial cells were harvested during the mid-exponential phase (OD_600_ = 0.6) for RNA extraction. The analysis showed a total of 68 significantly deregulated genes (log_2_FC > |1|, *adj p* < 0.01) in the Δ*rny* strain compared to the wild type, with 26 genes downregulated (including the residual *rny* gene) and 42 genes up-regulated (Figure 4C). Among the upregulated genes in the Δ*rny* strain, a cluster of 6 genes, including the chaperonin-encoding *groEL*, belongs to the “post-translational modification, protein turnover, chaperones” category. Moreover, the expression of several representatives of Hop and Hop-related (Hor) family of *H. pylori* outer membrane proteins, involved in gastric colonization and host-pathogen interaction, resulted significantly increased in the Δ*rny* strain, with a few of them with a log_2_FC just below the threshold: *hopA*, *horL*, *horE* (log_2_FC = 0.91), *hopQ*, the sialic acid binding protein *sabA*, and the adherence associated proteins *alpA* and *alpB* (log_2_FC = 0.98).^48^^,^^49^^,^^50^ In addition, at least other 4 genes coding for structural (*flaB* and *flgE*) or regulatory (*fliK*) flagellar proteins and the urease subunit β (*ureB*) were upregulated upon RNase Y depletion. The transcriptome analysis further revealed that the expression of ncRNAs overall was not affected in the Δ*rny* strain when compared to the wild type. Noteworthy, the expression level of only the CncR1 sRNA was significantly increased in the Δ*rny* strain, likely suggesting the specific involvement of the RNase Y in the post-transcriptional regulation of such sRNA.

To further investigate the possible role exerted by RNase Y in the posttranscriptional regulation and metabolism of sRNAs, we assessed the expression of *cncR1*, *repG*, and *nikS* transcripts (3 well-characterized sRNAs of *H. pylori*) by means of quantitative real-time PCR (RT-qPCR). It is worth highlighting that the RNA-seq analysis indicates the upregulation of CncR1 in the Δ*rny*, while RepG and NikS sRNAs do not belong to the list of deregulated genes in the Δ*rny* strain (Table S5). In order to assess the specificity of the RNase Y-driven regulation, we included in this analysis two complemented strains generated from an *rny*-null background: Δ*rny*::*rny* (ectopically expressing the wild-type *rny* sequence) and Δ*rny*::*rny*_H374A_ (carrying an H374A point mutation in the ectopic *rny* sequence). In both mutants, the chimeric construct made of P_3705_ promoter and *rny* CDS was cloned in the *vacA* locus. Results shown in Figure 4D reveal a significant 6.3 increase in the transcript levels of *cncR1* in the Δ*rny* (light gray bars), compared to the wild type strain (black bars). Moreover, the *in trans* complementation of the knock-out mutant with the wild type *rny* gene locus (Δ*rny*::*rny* strain) restored the expression level of *cncR1* to wild-type levels (dark gray bar). Interestingly, the Δ*rny*::*rny*_H374A_ strain, expressing a mutated RNase Y protein, showed an expression pattern similar to that observed in the knock-out (8.6-fold increase in the transcript levels of *cncR1*, with respect to the wild-type strain, Figure 4D, striped gray bars). These results parallel with the growth retardation observed in the Δ*rny*::*rny*_H374A_ strain (generation time = 3.39 ± 0.12 h) (Figure 4B) and collectively sustain the hypothesis that His residue in the HD catalytic domain is likely fundamental for the phosphohydrolytic activity of the protein. Nonetheless, such a regulatory pattern was not detected for *repG* or *nikS*, which showed invariant levels among wild type, knock-out, complemented, and H374A complemented strains.

These results, in addition to validate the data obtained from RNA-seq analysis, indicate that RNase Y seems not to be involved in regulating the metabolism of all transcripts belonging to the class of sRNAs, but rather to act on specific targets, including the noncoding RNA CncR1.

### RNase Y is involved in the metabolism of CncR1

The RNase-mediated processing of ncRNAs is likely crucial for the maturation. Eventually, the function of these regulators and examples of the involvement of RNases in the metabolism of different classes of sRNA molecules have been reported.^51^

**Figure 5:**
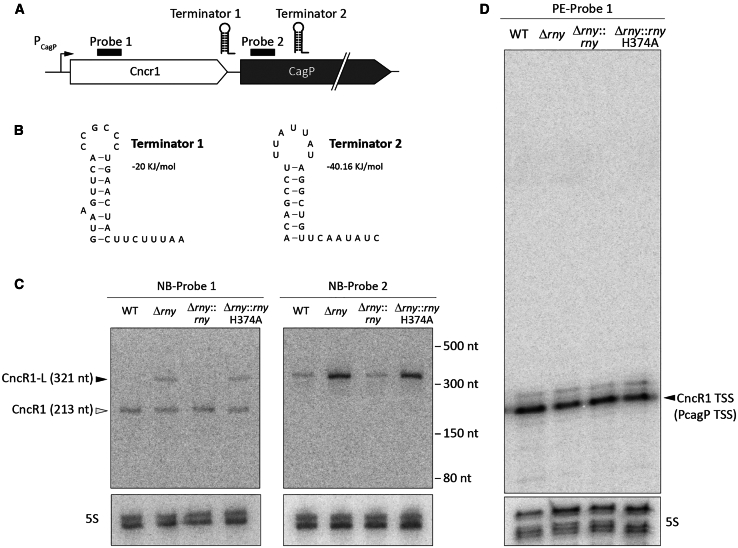
RNase Y regulates the different Cncr1 isoforms (A) Schematic representation of *cncR1* gene locus in *H. pylori* G27 wild type strain with the primary *cncR1* terminator (terminator 1) and the alternative predicted one (terminator 2). Probes used in northern blot and primer extensions analyses are represented as black boxes. (B) Bioinformatic prediction of the terminator 1 and terminator 2 secondary structures by RNA-fold. (C) Northern blot analyses of *cncR1* transcript in G27 wild type, Δ*rny*, Δ*rny*::*rny*, and Δ*rny*::*rny*_H374A_ strains. Oligonucleotides 536pe17 (probe 1) and 536pe20 (probe 2) were employed for the analysis, mapping in the 5′-region of *cncR1* transcript and downstream the terminator 1, respectively. Probe 5S-F annealing to the 5S transcript was included as a loading control. White and black triangles indicate CncR1 (213 nt) and CncR1-L (321 nt) transcripts, respectively. (D) Total RNA extracted from G27 wild type, Δ*rny*, Δ*rny*::*rny*, and Δ*rny*::*rny*_H374A_ strains was subjected to primer extension analysis with oligonucleotide 536pe17 (probe 1), showing the CncR1 TSS (identical to PcagP TSS, mapped in the study by Vannini A. et al.^52^). Parallel reactions performed with 5S-F oligonucleotide were used as loading controls.

Based on these observations, we investigated the potential involvement of RNase Y on CncR1 maturation and processing by performing Northern blot experiments on total RNA samples extracted from *H. pylori* G27 wild type, Δ*rny*, Δ*rny*::*rny*, and Δ*rny*::*rny*_H374A_ cultures. The hybridization with the “probe 1” (oligonucleotide 536pe17), mapping in the 5′ region of CncR1 (Figure 5A), revealed the presence of the primary *cncR1* transcript (213 nt) in all the tested strains (Figure 5C). Intriguingly, an additional transcript with higher molecular weight (>300 nt) specifically accumulates in the Δ*rny* and Δ*rny*::*rny*_H374A_ strains (expressing a catalytically inactive RNase Y). The same experiment carried out with the “probe 2” (oligonucleotide 536pe20), located downstream the CncR1 terminator, allowed the specific detection of the longer product (from here on named CncR1-L) both in the wild type and in the three mutant strains. By pixel densitometric analysis of this last image, CncR1-L was found to be 4.1- and 3.6-fold more abundant in Δ*rny* and Δ*rny*::*rny*_H374A_ mutants, respectively, compared to the wild type.

To get insights into the nature of the extended *cncR1* transcript, we looked for alternative TSS through primer extension experiments on RNA isolated from wt, Δ*rny*, Δ*rny*::*rny*, and Δ*rny*::*rny*_H374A_ samples. Extensions with probe 1 revealed that the *cncR1* TSS is not altered in an RNase Y-deficient background (Figure 5D). Indeed, the 5′-end of the *rny* transcript in the Δ*rny* and Δ*rny*::*rny*_H374A_ mutant strains was identical to that mapped in the wild type and Δ*rny*::*rny* strain and reported in the study by Vannini A et al.,^52^ and no additional bands corresponding to alternative TSSs were detected. These results may suggest that the longer *cncr1* isoform is due to differences in the 3′-end of the transcripts.

Then, to identify a possible terminator of transcription, responsible for terminating the CncR1 longer transcript, the RNAfold bioinformatic tool^53^ was used to predict a possible secondary structure of the RNA region downstream of the *cncR1* known terminator. Interestingly, this analysis revealed the presence of a stable RNA stem-loop structure (−40.16 kJ/mol) (Figure 5B) encompassing the positions 300–321 from the *cncR1* TSS, i.e., being properly positioned to terminate the CncR1-L transcript. However, when we analyzed the 20 nt downstream of the predicted stem-loop structure, we were unable to detect any T-stretch typical of the Rho-independent terminators of transcription. Nevertheless, the Rho-independent termination in *H. pylori* seems to adopt less stringent criteria compared to the canonical rules designed on the archetype *E. coli*.^54^ Of note, the predicted alternative *cncR1* terminator maps within the *cagP* CDS and no bands corresponding to a full length *cagP* transcript were revealed in our northern blot analysis. This observation aligns with previous studies suggesting the loss of expression of a prematurely truncated CagP open reading frame (∼600 nt) in *H. pylori* G27 ^55^. Hence, CncR1-L likely corresponds to the transcript arising from the invariant *cagP* TSS and terminating ∼321 nt downstream the TSS by the stem-loop detected at this position. Quantitative RT-PCR analysis at the P1, P2, and P3 regions of the *cncR1-cagP* transcript (Figure S6B) confirmed high expression levels upstream of terminator 1 (i.e., the transcription terminator identified in the study by Vannini A. et al.^55^), 10-fold reduction of transcript levels between the two terminators, indicating readthrough beyond terminator 1, and a further 10-fold reduction downstream of terminator 2 (i.e., the terminator pinpointed previously), corresponding to the readthrough transcript past this terminator. This latter transcript corresponds to the *cncR1-cagP* mRNA, while P2 detected CncR1-L and *cncR1-cagP*, and P1 measured CncR1-S, CncR1-L, and *cncR1-cagP*. Interestingly, these results were also observed in *H. pylori* P12 strain, which is predicted to express a full-length CagP protein (Figure S6C). Quantitative RT-PCR also confirmed the accumulation of P1 (corresponding principally to CncR1-S) in Δ*rny* mutants of both *H. pylori* G27 and P12 strains (Figure S6D) and an even higher accumulation of P2 (corresponding principally to *cncR1-L*) in the Δ*rny* mutants (Figure S6E). Hence, we hypothesized that a functional RNase Y may process CncR1-L isoform to nearly undetectable levels, thus leading *cncR1* shorter isoform to be the primary product generated from the P_*cagP*_ promoter.^55^

### CncR1 and CncR1-L have different affinities for *fliK* mRNA

**Figure 6:**
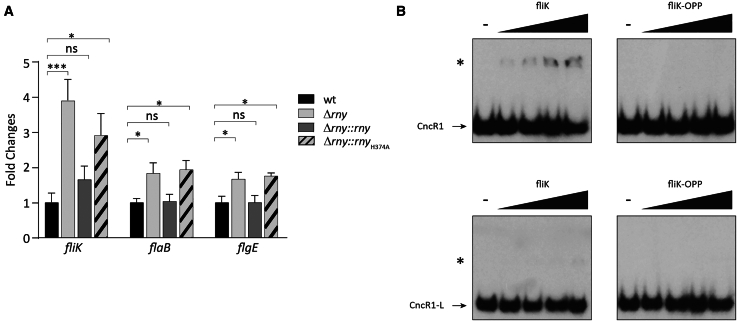
Dysregulation of CncR1 Metabolism in *Δrny* Impacts the Expression of CncR1 Targets (A) Quantitative RT-PCR (RT-qPCR) analysis of *fliK* (HPG27_RS04430), *flaB* (HPG27_RS00600) and *flgE* (HPG27_RS04255) mRNA levels in Δ*rny* (light gray bars), Δ*rny*::*rny* (dark gray bars) and Δ*rny*::*rny*_H374A_ (striped gray bars) mutants compared to wild type (black bars) strain. Gene specific oligonucleotide pairs Flik5-RTF/Flik5-RTR (*fliK*), 115-RTF/115-RTR (*flaB*) and 870-RTF/870-RTR (*flgE*) were used. Ct values were normalized on the 16S internal control and gene expression is reported as n-fold variation to the wild type strain. Mean values from at least three biological replicates are reported, ±SEM. Statistical significance was calculated by one-way ANOVA test (multiple comparisons vs. wt) and expressed as: ns = not significative; ∗ = *p* value < 0.05; ∗∗∗ = *p*- value < 0.001. (B) Electrophoretic mobility shift assay (EMSA) with *in vitro* synthesized radiolabeled *cncR1* and *cncR1*-L RNA probes and *fliK* RNAs: full length *fliK* and its reverse complement *fliK-*OPP used as negative control. Fifty fmol of 5′-end labeled CncR1 (upper panel) or CncR1-L (lower panel) were allowed to interact with 0, 50, 100, 200, and 400 fmol of *fliK* and *fliK*-OPP in presence of yeast tRNA as a non-specific competitor. The samples were resolved on a native 4% polyacrylamide gel and exposed for autoradiography. Black arrows indicate free *cncR1* or *cncR1*-L radiolabeled probes, while slower migrating bands corresponding to probe-target complexes are indicated by an asterisk. The images are representative of three independent experiments.

Results presented earlier show that the inactivation of the RNase Y leads to the accumulation of a longer isoform of CncR1, which coexists in the cell together with the primary 213 nt-long *cncR1* transcript. To gain insights into the effects of CncR1 de-regulation on its targets, we determined the transcript levels of well-characterized regulatory targets of CncR1 in Δ*rny*, Δ*rny*::*rny*, and Δ*rny*::*rny*_H374A_ mutants with respect to the wild-type strain. Since it has been shown that CncR1 is involved in controlling flagellar assembly and cell motility,^55^ we focused our analysis on the *fliK* (HP0906), *flaB* (HP0115), and *flgE* (HP0870) genes. The results of the RT-qPCR validated the differential expression observed in the RNA-seq analysis, revealing an increased transcription of all the targets in the Δ*rny* and Δ*rny*::*rny*_H374A_ strains compared to the wild type (Figure 6A). The effect of *rny* deletion on *fliK* mRNA levels was confirmed also in *H. pylori* P12 strain (Figure S6E). The complementation of the *rny* sequence in the Δ*rny*::*rny* strain resulted in a recovered phenotype and restored the levels of the *fliK*, *flaB*, and *flgE* transcripts to those of the wild type. Previous analyses reported that CncR1 negatively regulates genes coding for regulatory and structural flagellar proteins, including *fliK*, *flaB*, *flgE*, and the genes within their operons. The increases in transcript levels observed in the RNase Y deficient and inactive strains contrast the observed augmented *cncR1* transcription (see previous paragraph). In other words, the accumulation of CncR1 transcript observed in the Δ*rny* strain (Figure 4D) does not lead to a hyper-repression of CncR1 negatively regulated targets (*fliK*, *flaB*, and *flgE*) but instead to an accumulation of these transcripts (Figure 6A).

To shed light on this discrepancy, we assessed the capacity of both CncR1 isoforms to form stable complexes with the previously characterized CncR1 direct target (i.e., *fliK* mRNA) by RNA-RNA electrophoretic mobility shift assays (EMSA). *In vitro* synthesized 5′ end-radiolabeled CncR1 and CncR1-L probes were incubated with increasing concentrations of full-length *fliK* mRNA and its reverse complement *fliK*-OPP synthesized on the minus strand (negative control). As already demonstrated by Vannini and co-workers,^51^ when CncR1 was assayed with wild-type *fliK* mRNA, a slower migrating band appeared even at the lowest concentration tested (50 fmol, 1:1 M ratio), suggesting the formation of high affinity RNA-RNA complexes (Figure 6B, upper left panel). In contrast, CncR1-L revealed a nearly complete loss of affinity for *fliK*, as only a faint band appeared at the highest concentration tested (400 fmol), indicating a reduction in affinity that can be estimated to be approximately one order of magnitude, and thereby an impaired stability of the sRNA-mRNA interaction (Figure 6B, bottom left panel). No RNA duplexes were observed when both CncR1 and CncR1-L were incubated with the negative control *fliK*-OPP (Figure 6B upper and bottom right panels). Taken together these data suggest that CncR1-L may lose the capacity to form stable interactions with its targets and, therefore, to modulate their expression at a post-transcriptional level.

## Discussion

The streamlined and efficient signaling network of the human pathogen *H. pylori* is mainly responsible for its ability to adapt to its restricted host-associated environment (i.e., the gastric niche) and to generate persistent infections, while evading the host immune response.^56^ Regardless of the remarkably low number of 17 transcriptional regulators (including the vegetative σ^80^ and the two alternative σ^54^ and σ^28^ sigma factors), in the last few years several studies pinpointed the potential offered by the asRNA and sRNA in the post-transcriptional orchestration of gene expression in response to stress conditions, such as oxidative stress, acid pH, nickel starvation, and antibiotics.^57^^,^^58^ Their deletion or improper expression often led to severe phenotypes with increased susceptibility to antibiotics, hampered motility and reduced adhesion to host cells.^59^ For example, the *trans*-acting sRNA NikS inhibits the translation of the carcinogenic oncoprotein CagA, the vacuolating toxin VacA, and a spectrum of other outer membrane proteins by binding to the 5′UTR of their relative transcripts.^59^^,^^60^ In this perspective, the control over the decay and maturation of different RNA classes is of primary importance to ensure the maintenance of cellular homeostasis. Recent studies regarding the RNA degrading machinery in *H. pylori* highlighted the role of RNase J, as the primary participant in the initiation of bulk mRNA and asRNAs decay, and RNase III and RNase R, as involved to different extents in the maturation of stable rRNAs.^18^^,^^19^^,^^20^ With the present study, we provide for the first time a functional characterization of the single-strand specific endo-RNase Y in *H. pylori* G27, highlighting its putative involvement in the metabolism of a known sRNA (i.e., CncR1).

The RNase Y in *H. pylori* G27 strain is encoded from the *rny* gene. In accordance with the polycistronic organization revealed by the primary transcriptome analysis of the 26695 strain,^37^ our analysis of the *rny* genomic locus showed that this gene is transcribed in tandem with *HPG27_RS03705* from the P_3705_ promoter (Figure 1B). The operon is upregulated by Fur in response to iron depletion^38^ and downregulated during bacterial growth (Figure 2), indicating that its mRNA levels are finely tuned under varying environmental conditions encountered by the bacterium. The biological significance of these regulations needs further investigation.

The RNase Y has shown not to be essential for *H. pylori* viability in the tested conditions. In contrast to *B. subtilis* and *C. perfringens*, where the elimination or the depletion of RNase Y-encoding gene resulted in an important growth retardation,^32^ in *H. pylori* G27 the Δ*rny* mutant has shown only a moderate impact on the *H. pylori* fitness compared to the wild type, causing only a ∼30% (from 2.75 h to 3.57 h) retardation on *H. pylori* doubling time (Figures 4A and 4B). Similarly, *rny* deletion in *H. pylori* P12 was feasible and led to a similar phenotype, with reduced growth rate with respect to the parental strain (Figure S6A). Similar growth defects were also obtained in *S. pyogenes* and in *S. aureus* compared to the wild type.^35^^,^^61^ Interestingly, *H. pylori* strains expressing an RNase Y mutated in its predicted catalytic site (Δ*rny*::*rny*_H374A_) showed a phenotype which closely resembles the knock-out strain (Figures 4A and 4B). Also, in the Δ*rny*::*rny*_H374A_ strain, the transcript levels of the DEGs validated by qRT-PCR (i.e., *fliK*, *flaB*, *flgE*, and *cncR1*) were similar to those in the Δ*rny* strain (Figures 6A and 4D). This evidences the crucial role of the His374 residue for the phosphohydrolytic activity of the RNase Y and suggests that the observed effects in the Δ*rny*::*rny*_H374A_ mutant are due to the production of an inactive RNase Y. Similar results were observed in *B. subtilis*, where mutations in the metal-chelating HD domain hampered the RNase Y’s ability to process a riboswitch located in the *yitJ* leader region.^29^ Accordingly, the histidine residue at position 374 and aspartate at position 375 are strictly conserved in *H. pylori* and non-*H. pylori* orthologues (Figure S1B), along with a high degree of conservation of the entire KH ssRNA-binding and the HD catalytic domains. In contrast, the N-terminal region of the protein appears to be unique to the *H. pylori* specie (Figure S1B).

As revealed by the transcriptome analysis, the deletion of the RNase Y in *H. pylori* G27 triggered changes in the transcript levels of only 68 genes (Table S5). Among these, genes encoding important virulence factors involved in gastric environment adaptation (*ureB*) host-pathogen interaction (*sabA*, *hopA*, *horL*, *alpA*, *alpB*, and *hopQ*), motility (*flaB*, *fgE*, and *fliK*), and chaperones (*groEL*) were upregulated. In contrast, outer membrane proteins (*hopM*, *hofH*, and *HPG27_RS00420*) involved in the interaction with the host cell and antibiotic resistance resulted downregulated in the Δ*rny* mutant.^62^^,^^63^ These results suggest that RNase Y functions within a regulatory network, balancing the expression of specific virulence factors—enhancing the expression of some while downregulating others—either directly or indirectly, such as through CncR1, which is involved in the regulation of both motility and adhesion.^55^ Downregulated targets could result from additional direct RNase Y regulations that were not identified in this study (i.e., increased stability of targeted RNAs in the Δ*rny* mutant), from indirect regulation, as seen with CncR1, which has been shown to oppositely regulate different targets, or due to compensatory balance responses to the variations of gene expression. Such mechanisms have been observed in other bacterial systems, where RNase Y influences gene stability and expression in complex regulatory networks (e.g., *S. pyogenes* and *E. coli*).^6^^,^^23^ Notably, about one-third (23/68) of the genes deregulated in the Δ*rny* mutant intersects with the iron-dependent HpFur regulons,^38^ suggesting that RNase Y or its regulated targets may mediate some previously observed indirect effects. The small RNase Y regulon is consistent with the fact that RNase Y is non-essential in laboratory bacterial cultures grown in standard conditions. Such a modest influence on global transcript levels upon RNase Y depletion has already been documented in *S. pyogenes*, where this enzyme is primarily responsible for the stabilization of transcripts.^34^^,^^61^^,^^64^ However, examining the RNase Y regulon in animal models or under non-standard growth conditions (e.g., environmental stress) may reveal additional phenotypic effects due to the loss the enzyme. The RNase Y-mediated regulation of adhesins, its effects on the Fur regulon, and its role in *in vivo* models warrant further studies.

As previously speculated by the group of Tejada-Arranz, the inner membrane localization of the enzyme degrading machinery could reflect the turnover of specific subcategories of genes.^17^ CncR1 is the only *trans*-acting sRNA with regulatory function represented in our transcriptome analysis. In our laboratory, CncR1 was characterized for the first time as a 213 bp sRNA arising from the P_*cagP*_ promoter and whose transcription is phase-of-growth controlled by the essential master regulator HP1043 ^55^. CncR1 was demonstrated to be involved in modulating *H. pylori* virulence through opposite effects on motility and adhesion to host cells. The 3.34-fold increase of CncR1 transcript in the Δ*rny* strain contrasts with the overexpression of motility genes, as it was shown to negatively correlate with genes involved in flagellar assembly and biogenesis, including *fliK*, *flaB*, *flgE*, and *flgB*.^55^ Further, we demonstrate that in strain carrying a deletion for the RNase Y (*Δrny*) or expressing an inactive enzyme (*Δrny*_H374A_), a secondary transcript arising from the same CncR1 locus accumulates in the cell (Figure 5C). Taking advantage of RNAfold bioinformatic tool and northern blot experiments performed on the 3′ region downstream of the known CncR1 terminator, we revealed that this secondary transcript (which we called CncR1-L) terminates at a secondary strong (−40.16 kJ/mol) terminator sequence located ∼320 bp after the CncR1 transcription start site (Figure 5B). Although the presence of an alternative transcript (CncR1-L) from the P*cagP* promoter has already been proved in wild-type conditions, it was considered a barely detectable readthrough or unprocessed product.^55^ In addition, the CncR1-L transcript was not capable to form stable complexes *in vitro* with the *fliK* mRNA compared to CncR1 (Figure 6B).

These evidences, in addition to the involvement of RNase Y in the maturation and processing of different RNA molecules in other pathogens,^29^^,^^31^ allowed us to speculate that the RNase Y acts in order to degrade the CncR1-L readthrough product or to process the CncR1-L to obtain a mature CncR1 molecule. The hypothesis of CncR1-L as a CncR1 precursor would agree with the increased expression of flagellar genes observed in both *ΔcncR1* and *Δrny* strains. More in detail, data presented in this work indicate that, while in the wild-type strain the *cncR1* short transcript is highly predominant over CncR1-L (Figure S6C), in the *Δrny* strain we observed an accumulation of the long readthrough isoform. Although the CncR1-L isoform does not accumulate to levels exceeding those of CncR1-S (Figure 4C), its presence is associated with the deregulation of target transcripts (Figure 6A). One possible interpretation of these data suggests that the simultaneous presence of a mixed CncR1 and CncR1-L population may influence the overall number and strength of the mature CncR1-target RNA interaction, thus impairing the post-transcriptional repression of motility genes. However, understanding the mechanism behind the apparent interference of the CncR1(L) isoform on the functionality of CncR1(S) requires further study. The processing of a single-strand endo-RNase is frequently needed for maturation of IGRs encoded sRNAs to reveal the seed region, increase their stability, or even produce two independent sRNA molecules with different regulons.^65^^,^^66^ Despite this, it remains to be understood whether the RNase Y catalytic activity could be related to CncR1-L in a sequence- or structure-specific manner. In *S. aureus* and in *S. pyogenes* the analysis of the 5′ end of the processed transcripts generated following RNase Y cleavage revealed the presence of a G located upstream of the processing site in 58% and 87.4% of target RNAs, respectively.^34^^,^^61^ However, this prerequisite was not reported in *B. subtilis*. Indeed, in this bacterium and *S. aureus*, RNase Y seems sensitive to the RNA secondary structure downstream of its cleavage site more than to a nucleic acid sequence motif.^29^^,^^33^^,^^35^

### Limitations of the study

In this study, we have successfully characterized multiple facets of the RNase Y protein in *H. pylori*, which, until now, has been categorized solely based on the identification of conserved catalytic domains. A particularly intriguing aspect of this ribonuclease is its role in processing CncR1, a regulatory RNA associated with pathogen virulence. The presented data demonstrate that the inactivation of RNase Y results in the *in vivo* accumulation of a 3′ extended isoform of CncR1. Furthermore, our observations indicate that the extended isoform of CncR1 is incapable of interacting with *fliK* mRNA *in vitro*, a known regulatory target from previous studies. Regrettably, the available data only allow speculation about the specific action of the ribonuclease on CncR1. We remain uncertain whether RNase Y functions to entirely degrade the extended (non-functional) isoform of CncR1 or processes the extended isoform to generate the functional transcript, which is 213 nucleotides long. Detailed molecular analysis was hindered by the challenge of obtaining purified recombinant protein for *in vitro* assays. Despite numerous attempts to express and purify RNase Y using a variety of expression and purification conditions, the protein exhibited a high degree of insolubility under all tested circumstances. This insolubility was evident both when attempting to express the full-length isoform and when expressing a version lacking the N-terminal domain responsible for membrane association and coiled-coil formation. The development of a strategy for the expression and purification of RNase Y is crucial to enable a comprehensive biochemical-molecular characterization of this pivotal *H. pylori* enzyme.

## Resource availability

### Lead contact

Requests for further information and resources should be directed to and will be fulfilled by the lead contact, Prof. Davide Roncarati (davide.roncarati@unibo.it).

### Materials availability

Plasmids generated in this study are available from the lead contact (Prof. Davide Roncarati: davide.roncarati@unibo.it) with a completed materials transfer agreement.

### Data and code availability

Bam data have been deposited at Sequence Reads Archive (SRA) as BioProject PRJNA1182986 and are publicly available as of the date of publication.

## Acknowledgments

This research was funded by 10.13039/501100021856MUR, grant PRIN 2020YXFSW5 to V.S., and from Alma Mater Studiorum – 10.13039/501100005969University of Bologna to V.S. and D.R.

## Author contributions

Conceptualization, F.D’A., D.R., and V.S.; methodology, F.D’A., D.R., and A.V.; investigation, F.D’A., A.V., and A.M.; formal analysis, F.D’A. and E.P.; writing – original draft, F.D’A. and D.R.; writing – review and editing, F.D’A., A.V., E.P., V.S., and D.R.; visualization, F.D’A.; funding acquisition, V.S. and D.R.; supervision, V.S. and D.R.

## Declaration of interests

The authors declare no competing interest.

## STAR★Methods

### Key resources table

**Table:** 

REAGENT or RESOURCE	SOURCE	IDENTIFIER
**Antibodies**		

ANTI-FLAG® M2	Sigma-Aldrich	Cat# F1804; RRID: AB_262044
Anti-Hp1043	Pelliciari et al. 2017^67^	
Anti-BabA	Hansen et al. 2017^68^	

**Bacterial and virus strains**		

*E. coli* DH5α	Hanahan. 1983^69^	
*H. pylori* G27	Xiang et al. 1995^36^	
*H. pylori* P12	Fisher et al. 2010^70^	
*H. pylori* G27 Δ*rny*; bp from 135 to 1487 of the RNase Y (HPG27_RS03700) CDS replaced by a *km* cassette	This paper	
*H. pylori* G27 *Δrny::rny; rny* KO and with the chimeric construct made of P_3705_ promoter and *rny* CDS cloned in the *vacA* locus	This paper	
*H. pylori* G27 Δ*rny::rny*_H374A_*; rny* KO and with the chimeric construct made of P_3705_ promoter and *rny* CDS with the H374A mutation cloned in the *vacA* locus	This paper	
*H. pylori* G27 Δ*rny::rny-FLAG; rny* KO and with the chimeric construct made of P_3705_ promoter and *rny* CDS fused with the FLAG tag cloned in the *vacA* locus	This paper	
*H. pylori* P12 Δ*rny*; bp from 135 to 1487 of the RNase Y (HPG27_RS03700) CDS replaced by a *km* cassette	This paper	

**Chemicals, peptides, and recombinant proteins**		

N-lauroylsarcosine sodium salt	Sigma-Aldrich	L5777
Complete EDTA-free protease inhibitor	Roche, supplied by Sigma-Aldrich	04693132001
Yeast tRNA	Life Technologies	AM7119

**Deposited data**		

Raw and analyzed data	This paper	BioProject PRJNA1182986
*H. pylori* G27 RefSeq annotation GCF_000021165.1 sept-2022 release	https://www.ncbi.nlm.nih.gov/datasets/genome/GCF_000021165.1/	

**Oligonucleotides**		

See Table S1 for oligonucleotide list	This paper	Table S1

**Recombinant DNA**		

pBluescript KS II+	Stratagene	212207
pBS::cat; pBluescript KS II derivative carrying a HincII *Campylobacter colicat* cassette from pDT2548 cloned into the SmaI site of the vector	Roncarati et al. 2011^71^	
pNKO::KmR; Suicide vector	Pflock et al. 2005^47^	
pVAC::CAT; pVAC::Km derivative, carrying a BglII/BamHI cat cassette from pBS::cat	Pepe et al. 2018^72^	
pVAC::CAT-P3705- HPG27_RS03705-rny; pVAC::CAT derivative containing a 2518 bp XbaI/KpnI fragment amplified with oligos 716-F_XbaI and 717-R_KpnI on the *H. pylori* G27 chromosomal DNA	This paper	
pVAC::CAT-P3705-rny; pVAC::CAT::761-rny derivative obtained by whole around PCR with oligos Δ717wa_F and Δ717wa_R. HPG27_RS03705 harbours an in-frame deletion from bp 26 to 519 of its CDS	This paper	
pVAC::CAT-P3705-rnyH374A; pVAC::CAT::rny derivative obtained by mutagenesis PCR with oligos YH374A-F and YH374A-R	This paper	
pVAC::CAT-P3705-rny-FLAG; pVAC::CAT::rny derivative obtained by mutagenesis PCR with oligos Y_C-FLAG_Fw and Y_C-FLAG_Rev	This paper	

**Software and algorithms**		

Bowtie 2 (v2.2.6)	Langmead et al. 2012^73^	
BEDTools (v2.20.1∗)	Quinlan et al. 2010^74^	
SAMtools (v0.1.19)	Li et al. 2009^75^	
FeatureCounts	Liao et al. 2014^76^	
DESeq2 (v1.4.5)	Love et al. 2014^77^	
AlphaFold	Jumper et al. 2021^39^	https://alphafold.ebi.ac.uk/
RoseTTAFold	Baek et al. 2021^41^	https://robetta.bakerlab.org
DALI server	Holm et al. 2023^42^	http://ekhidna2.biocenter.helsinki.fi/dali/
InterPro	Paysan-Lafosse et al. 2022^43^	https://www.ebi.ac.uk/interpro/
RNAfold	Lorenz et al. 2011^53^	http://rna.tbi.univie.ac.at/cgi-bin/RNAWebSuite/RNAfold.cgi
MARCOIL	Delorenzi et al. 2002^44^	https://waggawagga.motorprotein.de/
Coiled-coils	Lupas et al. 1991^45^	https://npsa-prabi.ibcp.fr/cgi-bin/npsa_automat.pl?page=/NPSA/npsa_lupas.html
DeepCoil	Zimmermann et al. 2018^46^	https://toolkit.tuebingen.mpg.de/tools/deepcoil
GraphPad Prism 8.4.3		

### Experimental model and study participant details

#### *Helicobacter pylori* strains and growth conditions

Bacterial cells were recovered from frozen glycerol stocks on Brucella agar plates containing 7.5% of fetal calf serum (FCS) supplemented with Dent’s antibiotic mix. The plates were then incubated for 24–48 h at 37°C in a water-jacketed thermal incubator (9% CO_2_, 91% air atmosphere, and 95% humidity) or in jars using CampyGen (Thermo Fisher) atmosphere generation systems. Liquid cultures were grown in Brucella broth (BB) medium supplemented with 7.5% heat-inactivated FCS at 37°C with gentle agitation (140 rpm), in glass flasks until the indicated growth phase was reached. When required, antibiotics were added to solid media at the following concentrations: 30 μg/mL chloramphenicol and 25 μg/mL kanamycin.

### Method details

#### Construction of *H. pylori* mutant strains

Bacterial strains, plasmids, and constructs used in this study are reported in Key Resource Table and Table S1. All the *H. pylori* mutants were obtained by double homologous recombination of the naturally competent G27 or P12 clinical isolates as previously described.^36^^,^^70^ To generate the *H. pylori* G27 Δ*rny* strain, carrying the deletion of the *rny* gene, the G27 wild type strain was transformed with the G27-rny_UP-Km^R^-rny_DOWN PCR product obtained by a Double-Joint PCR procedure as described in Yu et al., 2004.^78^ Briefly, YKO-up-F/YKO-up-R-T and YKO-down-F-T/YKO-down-R couples of oligonucleotides (Table S1) were used to amplify the 520 bp up- and 573 bp down-stream homology regions flanking the *rny* gene from the G27 wild type genomic DNA (gDNA). The *aphA3* kanamycin resistance cassette was amplified with oligos KmF and KmR on vector pNKO::Km^R^. The three partially overlapping fragments were assembled and amplified with YKO-up-F/YKO-down-R oligonucleotides and 2 μg of the final purified PCR product were used to transform *H. pylori*. Similarly, *H. pylori* P12 Δ*rny* strain was obtained by transforming the P12 wild type strain with the P12-rny_UP-Km^R^-rny_DOWN PCR product obtained by Double-Joint PCR. To complement the *rny* sequence or its mutated variant *rny*_H374A_ in an *H. pylori* G27 *rny* knock-out (Δ*rny*) background, we selected the *vacA* gene locus as the target site for the insertion. 16-F_XbaI and 717-R_KpnI oligonucleotides were used to amplify a 2.5 kb fragment encompassing the whole *HPG27_RS03705-rny* ORF including its own promoter P_3705_. The PCR product was then purified and XbaI/KpnI inserted into pVAC::CAT, obtaining pVAC::CAT-P_3705_-*HPG27_RS03705*-*rny*. Then, *HPG27_RS03705* was removed from the latter DNA construct by a whole around PCR with oligos Δ717wa_F and Δ717wa_R, the product was ligated and transformed into *E. coli* DH5α.^69^ The ensuing pVAC::CAT-P_3705_-*rny* construct harbored an in-frame deletion of the gene, enabling us to complement the *rny* gene without introducing a second copy of a functional *HPG27_RS03705* gene. The plasmid pVAC::CAT-P_3705_-*rny*_H374A_, carrying the His to Ala mutation in position 374 of RNase Y CDS, was obtained by site directed mutagenesis using YH374A-F/YH374A-R primers on pVAC::CAT-P_3705_-*rny* template. Similarly, the plasmid pVAC::CAT-P_3705_-*rny*-FLAG, carrying the FLAG tag fused at the C-terminal of RNase Y, was obtained by site directed mutagenesis using Y_C-FLAG_Fw/Y_C-FLAG_Rev primers on pVAC::CAT-P_3705_-*rny* template. The constructs pVAC::CAT-P_3705_-*rny*, pVAC::CAT-P_3705_-*rny*_H374A_, and pVAC::CAT-P_3705_-*rny*-FLAG were used to generate *H. pylori* G27 Δ*rny*::*rny*, Δ*rny*::*rny*_H374A_, and Δ*rny*::*rny*-FLAG mutant strains, respectively.

#### DNA manipulations

DNA amplification, restriction digestions and ligations were performed following standard molecular procedures^79^ with enzymes purchased from New England Biolabs. Preparation of plasmid DNA were performed with NucleoSpin Plasmid (Macherey-Nagel) and DNA fragments purifications were carried out with NucleoSpin Gel an PCR Clean-up kit (Macherey-Nagel).

#### RNA isolation and RT-qPCR analysis

*H. pylori* liquid cultures were grown with gentle agitation (140 rpm) at 37°C in Brucella broth medium supplemented with 7.5% FCS. Cultures were harvested upon reaching the mid-exponential growth phase (OD_600_ = 0.6–0.8). For growth-phase expression analysis, cultures were sampled at specific phases: early exponential (OD_600_ = 0.3), mid-exponential (OD_600_ = 0.7), exponential-stationary transition (OD_600_ = 1.4), stationary (1 day), late stationary (1.5 days), and coccoid (4 days). For each sample, 10 mL of culture were mixed with 1.25 mL of ice-cold stop solution (95% ethanol +5% water-saturated phenol pH 4.5) and immediately spun down at 5,000 g for 10 min at 4°C. Dry pellets were stored at −80°C or directly resuspended in 1 mL of Tri-Reagent (Invitrogen). Total RNA extracts were isolated following the manufacturer’s procedure. To ensure RNA purity and integrity, an aliquot of each RNA sample was collected, quantified, and subjected to electrophoresis on a 1% agarose gel.

cDNA synthesis and RT-qPCR analysis were carried out as previously described.^72^ Briefly, prior reverse transcription, 2μg of RNAs were treated with RapidOut DNA Removal kit (Thermo Fisher Scientific) following manufacturer’s instructions, to eliminate residual genomic DNA. Then, 500 ng of RNA were mixed with 100 pmol of Random Hexamers (Invitrogen), denatured at 65°C for 5 min and rapidly chilled on ice. Reverse transcription was finally conducted with 200 U of RevertAid Reverse Transcriptase (Invitrogen) and dNTPs mix (1 mM each) at 25°C for 10 min, followed by 42°C for 60 min. RT-qPCRs were carried out as detailed in Pelliciari et al., 2015^80^ using PowerUp SYBR Green Master Mix (Applied Biosystems) with 50 pg of RNA per reaction. As a negative control, qPCR assays were conducted on samples in which the reverse transcription step was performed without adding the Reverse Transcriptase enzyme, along with qPCR ‘blank’ assays lacking template DNA. Ct values in the controls that were more than 5 cycles higher than those in qRT-PCR reactions using cDNA were considered acceptable. Relative gene expression was calculated using the ΔΔC_t_ method using the primers reported in Table S1 and normalized on the constitutively expressed 16S rRNA.

#### RNA-sequencing and data analysis

Total RNA was purified from *H. pylori* G27 liquid cultures of wild type and Δ*rny* strains as described in the previous paragraphs. Ribosomal RNA depletion, quality check and strand specific cDNAs library preparation were performed starting from 2 μg of total RNA through the Novogene company service. Each library was sequenced on an Illumina NovaSeq 6000 platform and 150 bp paired-end reads were produced. Bowtie 2 (v2.2.6)^73^ was used to align raw reads to *H. pylori* G27 genome. End-to-end mapping was performed and non-deterministic option was specified to force a single assignment of multi-mapping reads to the best scoring region (if present) or a random attribution in the case of regions with identical scores. High quality reads were selected requiring both read of the pair to be mapped, for uniquely mapping reads MAPQ (mapping quality) greater than 20, while for multi-mapping reads alignment score was set equal or greater than – 25. rRNA depletion, strand specificity, and gene coverage were evaluated using BEDTools (v2.20.1∗)^74^ and SAMtools (v0.1.19)^75^ to verify the library preparation and sequencing performances. FeatureCounts^76^ was adopted to produce raw counts requiring strand specificity of the read pair and -d 20 -D 1200 to include also reads with non-standard length of the pair occurring for example in tRNA and ncRNA transcripts. Read pairs overlapping multiple features were excluded. The R package DESeq2 (v1.4.5)^77^ was then used to normalize the counts and to individuate differentially expressed features showing BH (Benjamini-Hochberg) adjusted *p*-value lower than 0.01 and log_2_ Fold Change >|1|. COG annotation was obtained as described in^67^ and functional enrichments were estimated using Hypergeometric Test and BH correction for multiple testing.

#### *H.pylori* G27 manually curated genome annotation

*H. pylori* G27 RefSeq annotation (GCF_000021165.1) in the version released on sept-2022 was used as the reference for gene annotation to which we manually added validated ncRNAs (*anti_HsrA, cncr1, isoB, nrr1, nrr2, sRNA2*^55^^,^^67^). We also revised the annotation of protein coding genes that, based on our sequencing data, were improperly annotated as pseudogenes in this version of the reference genome (*babB, ureF, HPG27_RS01025, HPG27_RS01370, hopL, HPG27_RS00760, HPG27_RS01965, HPG27_RS02135, alaS, rpoB, uvrA, rpoA, rsmI, ubiA, HPG27_RS02165, rnpA, hspR, tonB, nuoM, tatA, zntA, dnaX, oppA, HPG27_RS07565, HPG27_RS01365*), indicating them as “protein-coding”. To obtain COG annotation for the putative protein coding targets, the protein accession number of those genes were submitted to CDD online database (sept-2022 release) and COG alphanumeric code was converted to function according to the official COG classification (sept-2022 release).

#### Primer extension analysis

Primer extension analyses were conducted following the procedure outlined in Roncarati et al.*,* 2011.^71^ Twenty μg of RNA were precipitated and resuspended in 10 μL of primer extension reaction mix containing 0.1 pmol of radiolabeled primer, 400 nM dNTPs, in 1x Revert Aid buffer. After 2 min of denaturation at 95°C, 200U of Revert Aid Reverse Transcriptase (Invitrogen) were added and the reactions were incubated at 42°C for 1 h. Afterward, the reactions were treated with 1 μL of RNase A (10 μg/μL, Sigma-Aldrich), subjected to phenol-chloroform-isoamyl alcohol (25:24:1) extraction, ethanol precipitated and resuspended in 7 μL of 95% formamide, 0.1% bromophenol blue, 10 mM EDTA pH 8.0 loading buffer. Samples were denatured at 100°C for 5 min, immediately chilled on ice, loaded on an 8M Urea, 6% Acrylamide/Bis (19:1) in 1x TBE buffer gel and run for 1h and 30 min at 42 W. Finally, gels were dried and autoradiographed for the visualization.

For the precise mapping of 5′-end of the *HPG27_RS03705*-*rny* transcript, a Sanger sequencing reaction was performed by using 717pe9 radiolabeled primer and the corresponding cloned P_3705_ promoter region.

#### Northern blot analysis

Table S1 reports the oligonucleotides used in the hybridization experiments. Five pmol of each primer was 5′-end radiolabeled with 6 pmol of [γ-^32^P]-ATP (PerkinElmer) with T4 polynucleotide kinase (New England Biolabs) at 37°C for 30 min. Bio-Spin Chromatography column (Bio-Rad) packed with Sephadex G-50 (Pharmacia Fine Chemicals) were used to remove the unincorporated radioactive nucleotide.

Northern blot analyses were carried out using 10 μg of total RNA extract separated under denaturing conditions in an 8M Urea, 6% Acrylamide/Bis 19:1 (Ambion) in 1x TBE buffer gel. Samples were then electroblotted to a Hybond-N+ nylon membrane (Amersham) in 1x TBE buffer at 50V for 1 h at 4°C and crosslinked to the filter by UV-ray treatment (5 J, λ = 356 nm). Membranes were prehybridized in 5 mL of ULTRAhyb Ultrasensitive Hybridization Buffer (Invitrogen) and subsequently hybridized overnight in the same buffer with 1 pmol of radioactively labeled probe at 42°C. Membranes were washed at 42°C with wash buffer I (5x SSC, 0.1% SDS), wash buffer II (1x SSC, 0.1% SDS), and wash buffer III (0.1x SSC, 0.1% SDS) for 15 min each. Radioactive signal was acquired with a Storm phosphor-imager (Amersham-GE) and quantified using the Image Quant Software (Molecular Dynamics).

#### Cellular fractionation, Western Blotg and LPS staining

The cellular fractionation protocol was adapted from Tejada-Arranz et al., 2020 and Cian et al., 2020.^17^^,^^81^ Briefly, *H. pylori* Δ*rny*::*rny*-FLAG liquid culture was grown up to mid-exponential phase (OD_600_ = 0.8) and 15 mL were harvested by centrifugation at 5,000 g for 8 min at 4°C. Cells were washed twice with PBS, resuspended in 1 mL of cold Lysis Buffer A (10 mM Tris-HCl, pH 7.4; 0.5 M sucrose; lysozyme 200 μg/mL; Complete protease inhibitor cocktail from Roche), and incubated on ice for 4 min. Then, 1 mL of 1.5 mM EDTA pH 8.0 was added to the sample and incubated on ice for 7 min. Bacterial cells were collected by centrifugation at 10,000 g for 5 min at 4°C, resuspended in 1.5 mL of cold Lysis buffer B (10 mM Tris-HCl, pH 7.4; 0.2 M sucrose; protease inhibitor), and disrupted by sonication with a Branson Digital Sonifier at 20% amplitude for 3 min (5 sec ON, 15 sec OFF) on ice. Cell debris was removed by centrifugation at 6,500 g for 10 min at 4°C, and the supernatant was collected as total extract (TE). 1.4 mL of TE were layered on 1.5 mL of 78% sucrose solution in a 4 mL ultracentrifuge tube (Beckman Coulter Life Sciences) and centrifuged at 140,000 g for 45 min at 4°C in an Optima L-90K ultracentrifuge using a 50.3Ti rotor (Beckman Coulter Life Sciences). The supernatants contained the cytosolic soluble (Cs) fraction, while the brown ring on the sucrose cushion was collected, diluted to 3 mL with Buffer B, layered to a new 78% sucrose cushion, and re-centrifuged in the same conditions. The brown ring corresponding to the total membrane fraction was diluted with lysis Buffer C (10 mM Tris-HCl, pH 7.4; protease inhibitor; 0.1% N-lauroylsarcosine sodium salt from Sigma-Aldrich) to a final volume of 1.5 mL. A few microliters were collected as the total membrane (Memb) fraction, while the remaining sample was incubated on ice for 30 min and then ultracentrifuged under the same conditions. The supernatant containing the inner membrane (IM) fraction was collected and the pellet was resuspended in 1.5 mL of buffer D (10 mM Tris-HCl, pH 7.4; protease inhibitor; 1% N-lauroylsarcosine sodium salt), incubated on ice for 30 min, and ultracentrifuged in the same conditions. The supernatant containing the outer membrane (OM) fraction was collected and diluted to 1.5 mL. Fifteen μL of each of the 5 cellular fractions were separated on a 12% SDS-PAGE gel and blotted to an Amersham Hybond PVDF membrane (GE Heathcare Life scince). Antibodies against FLAG tag (1 : 1,000; Sigma-Aldrich), Hp1043 (1 : 5,000; ^78^), and BabA (1 : 10,000; ^81^) proteins were used to detect Rny-FLAG, Hp1043, and BabA proteins, respectively. Goat α-rabbit IgG-HRP (Invitrogen) or goat α-mouse IgG-HRP (Sigma-Aldrich) were used as secondary antibodies at 1:10,000 dilution. The signal was acquired with a ChemiDoc MP Imaging System (BioRad). LPS silver-staining was adapted from Cian et al., 2020^81^ and Pernitzsch et al., 2021.^82^ Specifically, 15 μL of Cs, IM, and OM were treated with N-lauroylsarcosine sodium salt to a final concentration of 2%, 1 U DNase (RapidOut DNA Removal kit), and 10 μg RNase A, then incubated at 37°C for 50 min. The samples were then treated with SDS to a final concentration of 0.5% and 60 μg Proteinase K (Sigma-Aldrich) and incubated at 65°C for 120 min. Four μL of each sample were separated on a 15% SDS-PAGE, after which the gel was treated in fix solution (25% isopropanol; 7% acetic acid; 68% water) for 16 h, then treated with 0.7% NaIO_4_ in fix solution for 15min at 20°C, and washed 3 times with water for 30 min each. The gel was then incubated in silver-staining solution (0.35% NH_3_; 20 mM NaOH; 0.4% AgNO_3_) for 10 min, washed 3 times with water for 15 min each, incubated in developing solution (2.5% Na_2_CO_3_; 0.01% formaldehyde) for 30 min, and finally in stop solution (50 mM EDTA).

#### Structure prediction analyses

The AlphaFold (https://alphafold.ebi.ac.uk/) and RoseTTAFold (https://robetta.bakerlab.org) were used to obtain the structural model predictions for the RNase Y protein.

The Dali sever (http://ekhidna2.biocenter.helsinki.fi/dali/) was used to compare the RNase Y structural model generated by AlphaFold against protein structures in the Protein DataBank (PDB).

The InterPro (https://www.ebi.ac.uk/interpro/) predictor was used to obtain a comprehensive prediction of the functional domains of the RNase Y protein.

The RNAfold (http://rna.tbi.univie.ac.at/cgi-bin/RNAWebSuite/RNAfold.cgi) online program was used to predict the secondary structure of RNA sequences.

#### RNA probe preparation and RNA-RNA EMSA

The couple of primers (see Table S1) T7-cncR1_ivtF2/cncR1-term1_ivtR (*cncR1*-S), T7-cncR1_ivtF2/cncR1-term2_ivtR (*cncR1*-L), T7-fliK_ivtF2/Flik-ivtR (*fliK*) and FliKOPP-ivtF/T7-FliKOPP-ivtR2 (*fliK*-OPP) were used to amplify DNA templates from G27 wild type genomic DNA for the *in vitro* synthesis of the corresponding RNA transcripts. *cncR1*-S and *cncR1*-L probes were 5′-end labeled as in Vannini et al., 2016.^55^ Gel shift assays were performed using 50 fmol of 5′-end labeled *cncR1*-S or *cncR1*-L with increasing amounts (50, 100, 200 and 400 fmol) of purified *fliK* or *fliK*-OPP transcripts in 15 μL reactions. Briefly, labeled RNAs were denatured at 65°C for 5 min and then slowly cooled to 37°C for 10 min to allow renaturation with 300 ng of yeast tRNA (Life Technologies) as non-specific competitor in EMSA RNA buffer (10 mM Tris–HCl pH 7.0, 100 mM KCl, 10 mM MgCl_2_, 10% glycerol). Then renatured RNA putative targets were added, and the final reaction was kept for 15 min at 37°C to allow the formation of possible sRNA-target complexes. Binding reactions were stopped in ice and resolved on 4% polyacrylamide gels at 2 V/cm for 1.5 h at 4°C in Tris–borate buffer (60 mM Tris, 240 mM boric acid, pH 8.0). Dried gels were finally autoradiographed to acquire the radioactive signal.^55^

### Quantification and statistical analysis

Three to four independent replicates of bacterial cultures (biological replicates) were treated as described in the corresponding sections. Results are presented in the graphs as mean values ± Standard Deviation (SD) or Standard Error of the Mean (SEM). Statistical analyses were conducted using GraphPad Prism 8.4.3. Data were compared using two-way T-tests or ANOVA with multiple comparisons (either all comparisons or comparisons to the control sample). Normality was assessed using the Shapiro-Wilk test, and variance equality was evaluated with the F-test (for T-tests) or the Brown-Forsythe test (for ANOVA). Specific details of each assay are provided in the respective figure legends.

## Supplemental information

Document S1. Figures S1–S6 and Tables S1, S4, and S5

Table S2. Conservation in HP, related to Figures 3 and S1

Table S3. Conservation in Non-HP, related to Figures 3 and S1
