# Supplementary material for: RNase Y mediates posttranscriptional control of the virulence-associated CncR1 small-RNA in Helicobacter pylori
Source: iScience. 2025 Jan 16;28(2):111815. doi: 10.1016/j.isci.2025.111815 (PMC11821409; doi:10.1016/j.isci.2025.111815)
Supplement: Document S1. Figures S1–S6 and Tables S1, S4, and S5 [file mmc1.pdf]

iScience, Volume *28*

Supplemental information

RNase Y mediates posttranscriptional control of the virulence-associated CncR1 small-RNA in *Helicobacter pylori*

Federico D'Agostino, Eva Pinatel, Alexandra Meynhardt, Vincenzo Scarlato, Andrea Vannini, and Davide Roncarati

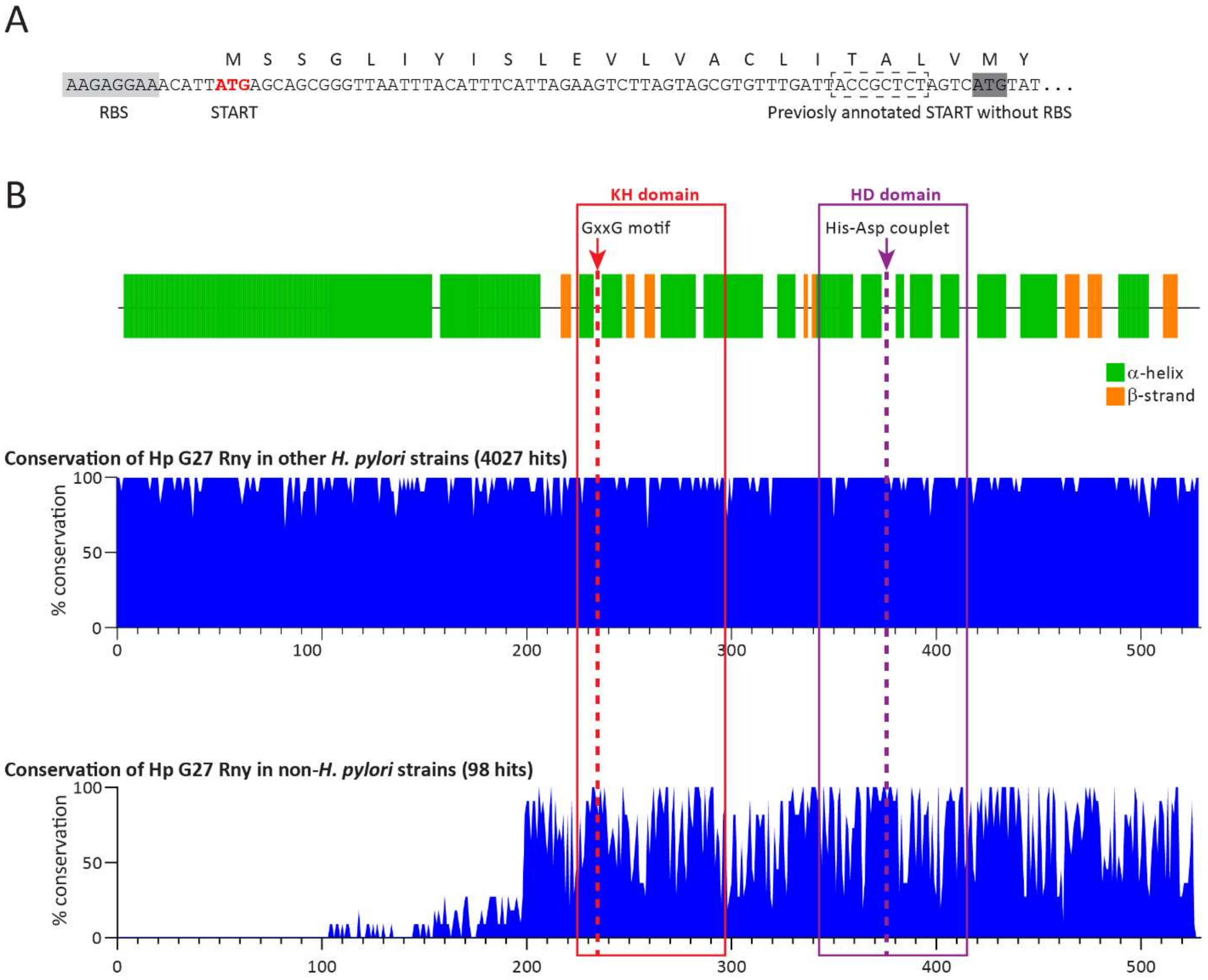

Figure S1: Features of *rny* transcript and aminoacidic conservation of Rny protein, related to: Bioinformatics analysis of the *H. pylori* RNase Y structure. A) Nucleotide sequence of the 5' region of *H. pylori* G27 (Hp G27) *rny* transcript, with the ribosome binding site (RBS) highlighted in a light grey box, and the start codon shown in red. The previously annotated start codon is highlighted in a dark grey box, with the absence of an upstream RBS noted by a dashed box. B) Secondary structure of Hp G27 Rny protein as predicted by AlphaFold. The graphs below display the percentage of amino acid conservation among orthologues identified via BLAST-P analysis, both within *H. pylori* strains (middle panel) and in non-*H. pylori* species (bottom panel). The KH and HD domains are highlighted in red and purple, respectively. The GxxG single-strand RNA-binding motif within the KH domain and the conserved His-Asp pair in the HD domain are marked by arrows and vertical dashed lines.

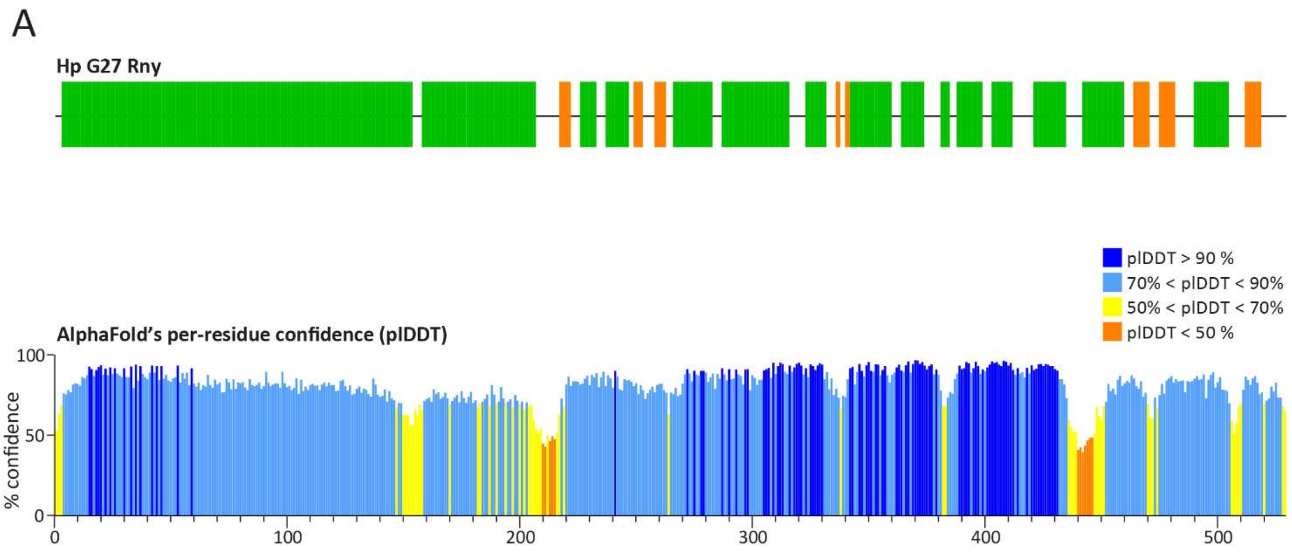

Figure S2: Confidence of the *H. pylori* G27 RNase Y model as predicted by AlphaFold, related to: Bioinformatics analysis of the *H. pylori* RNase Y structure. A) Secondary structure of the H. pylori G27 Rny protein model generated by AlphaFold. Below, the AlphaFold per-residue confidence scores (pLDDT) are shown, calculated as the mean confidence for all atoms within each amino acid residue. Confidence values are presented as histograms, color-coded according to defined ranges: higher scores, shown in blue and cyan, indicate more reliable predictions.

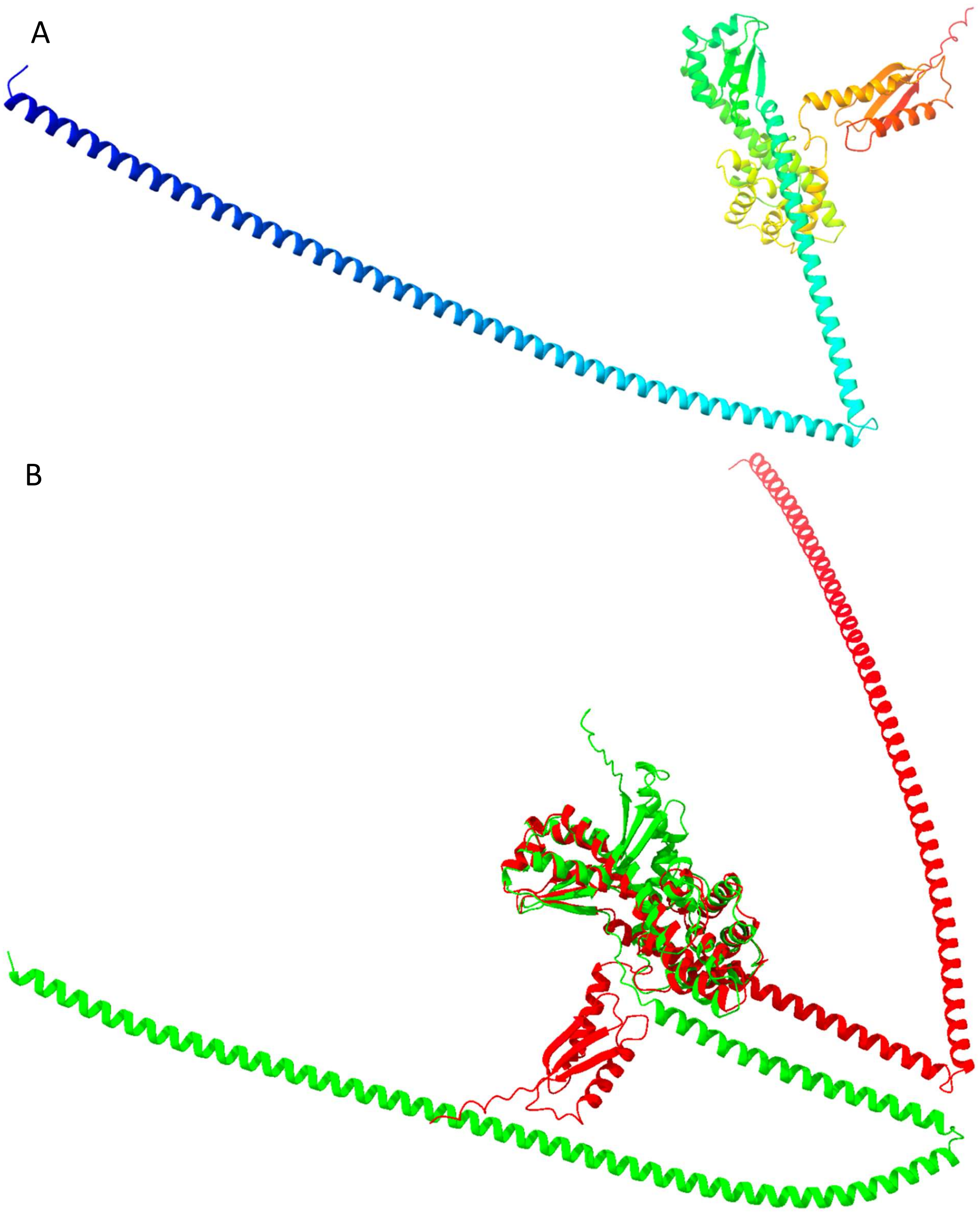

Figure S3: Model of Hp G27 RNase Y by RoseTTAFold, related to: Bioinformatics analysis of the *H. pylori* RNase Y structure. A) Three-dimensional model of Hp G27 RNase Y obtained using the RoseTTAFold tool. B) Superposition of the Hp G27 RNase Y AlphaFold (in green) and RoseTTAFold (in red) structural models.

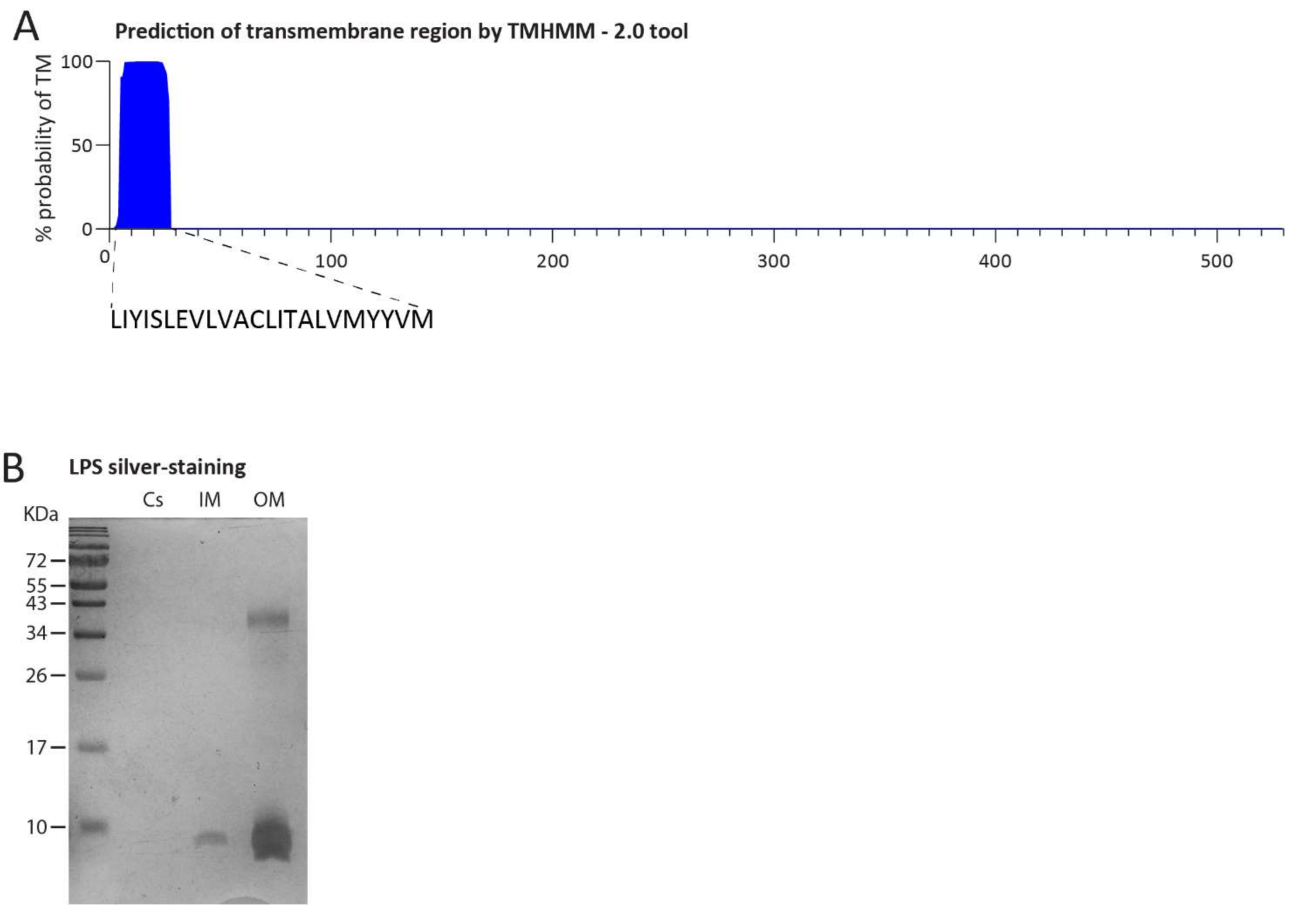

Figure S4: Hp G27 Rny protein topology and determination of LPS in cellular fractions, related to: Bioinformatics analysis of the *H. pylori* RNase Y structure. A) Prediction of the Hp G27 Rny transmembrane (TM) region using the TMHMM 2.0 tool indicates that amino acids 5 to 27 in the first α-helix of the protein are likely to span the membrane (the amino acid sequence of the predicted TM region is shown below). B) Analysis of LPS localization in the soluble (Cs), inner membrane (IM), and outer membrane (OM) fractions. Equal amounts of each fraction were processed as described in the Materials and Methods section, separated on a 15% SDS-PAGE gel alongside a protein marker for molecular weight determination, and visualized by silver staining.

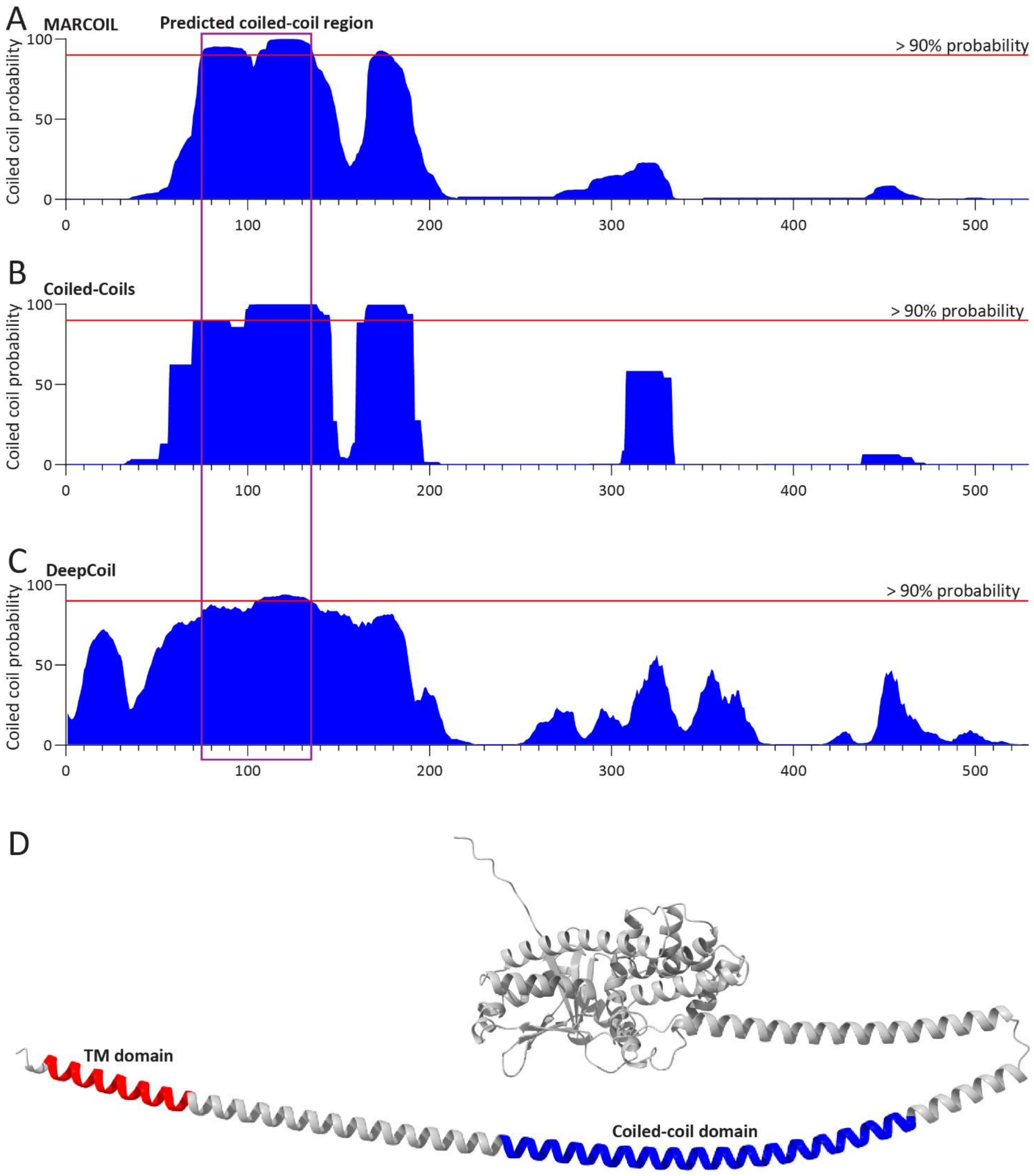

Figure S5: Prediction of a coiled-coil dimerization domain, related to: Bioinformatics analysis of the *H. pylori* RNase Y structure. A-C) Various bioinformatic tools were utilized to identify potential coiled-coil dimerization regions: A) MARCOIL[1]; B) Coiled-coils[2]; C) DeepCoil[3]. The region spanning amino acids 75 to 135 shows a high probability of forming a coiled-coil domain, potentially facilitating homo- or hetero-multimerization, as consistently predicted by all three bioinformatic tools. D) Hp G27 RNase Y structure as predicted by AlphaFold, showing the predicted TM domain in red and the coiled-coil domain in blue.

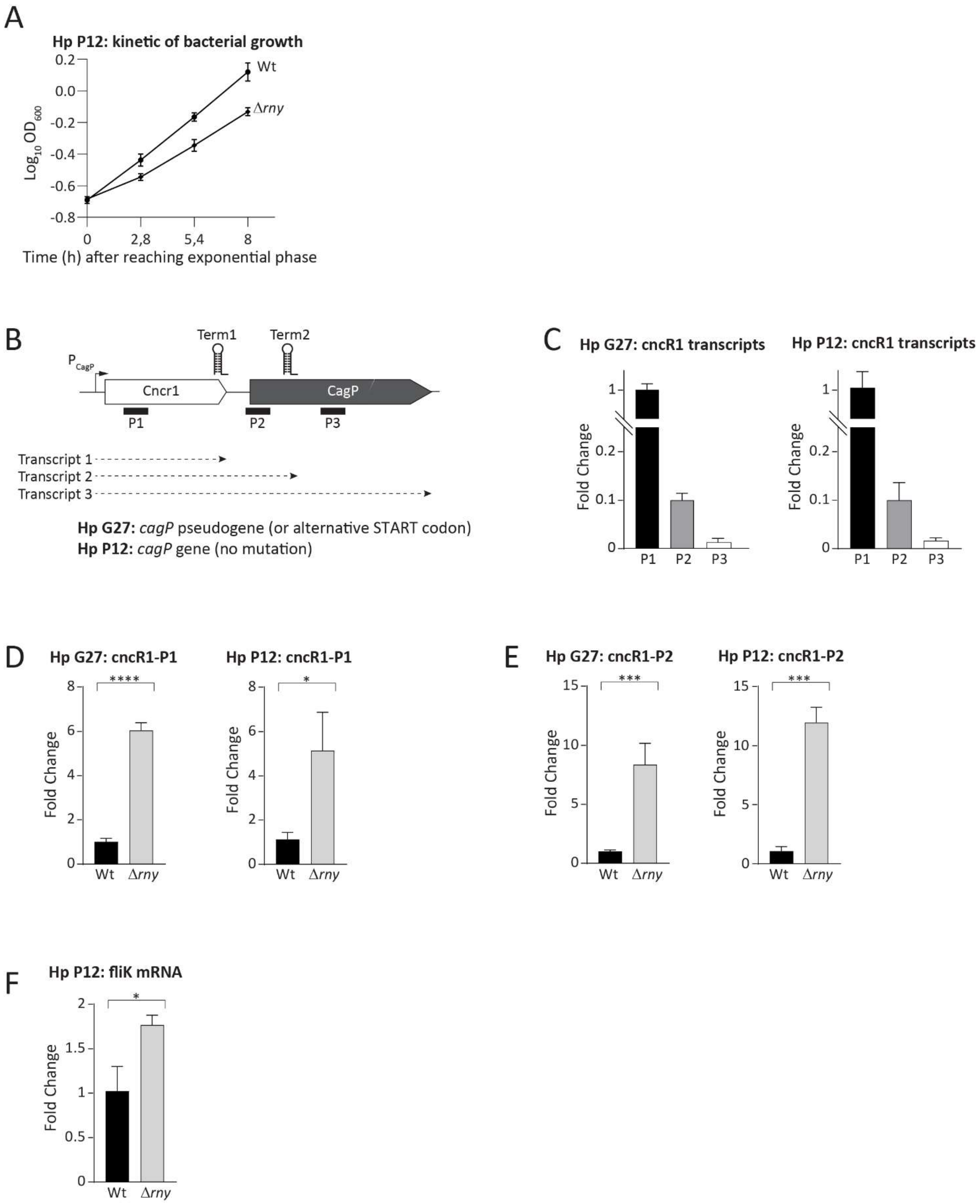

Figure S6: Analysis of Hp P12 *rny* deletion, related to: RNase Y is involved in the metabolism of CncR1. A) Growth kinetics of the Hp P12 wild-type (Wt) and Δ*rny* strains after reaching exponential growth. Mean values from 3 biological replicates are reported +/- SD. B) Schematic representation of the CncR1 locus in Hp G27, showing the TSS, *cagP* CDS, and two predicted terminator sequences. The 3 transcripts previously[4] detected: Transcript 1 (from TSS to terminator 1), Transcript 2 (from TSS to terminator 2), and Transcript 3 (from TSS to the end of *cagP*). Hp G27 *cagP* CDS is out of frame, but alternative start codons exist at the 5' end of the transcript. Hp P12 *cagP* CDS is predicted to produce a full-length CagP protein with 96% identity to Hp 26695 HP0536 ORF. C) Transcript levels in the CncR1 locus (P1, P2, and P3 regions) in Hp G27 and P12. Analysis was conducted with primers 536-RTF / 536-RTR for Hp G27 and 536RTR_P12 / 536RTF_P12 for Hp P12,

normalized to the 16S probe, and reported as fold change relative to P1. Data represent mean values from 3 biological replicates +/- SD. D) Transcript levels at the *cncR1* P1 region in wt and Δ*rny* strains of Hp G27 (536F5 / 536R5 primers) and Hp P12 (536F5 / 536R5_P12 primers). E) Transcript levels at the *cncR1* P2 region in wt and Δ*rny* strains of Hp G27 (536F6 / 536R6 primers) and Hp P12 (536F6 / 536R6_P12 primers). F) *fliK* transcript levels in Hp P12 wt and Δ*rny* strains, analyzed with primers Flik5-RTF / FliK5-RTR. (D-F) Ct values were normalized on the 16S internal control and gene expression is reported as n-fold changes with respect to the corresponding wt strain. Mean values from 3 biological replicates are reported +/- SD. The statistical significance was calculated with Unpaired two-tailed T-test and expressed as: * = $p < 0.05$, *** = $p < 0.001$, **** = $p < 0.0001$

Table S1: Oligonucleotides used in this study, related to: STAR Methods

Name	Description	Reference
YKO-up-F	ATCGCCACAGAATATGCGCATTTG	This study
YKO-up-R-T	CTAGATTTAGATGTCTAAAAAGCTTATCGATCGCTAAAAAGATAGAAGAGAGTGC	This study
KmF	ATCGATAAACCCAGCGAACCATTTG	This study
KmR	TCGATAAGCTTTTTAGACATCTAAATCTAG	This study
YKO-down-F-T	CCTCAAATGGTTCGCTGGGTTTATCGATTAGCTGAAGCACTTTTTAAAGTAGC	This study
YKO-down-R	TTAGGGCATGAGCTTGACATTAG	This study
716-F_XbaI	TATATCTAGAGGATCATGCCCATTTACTTGC	This study
717-R_KpnI	TATAGGTACCGTGGTATTGTTTTGAGAATCTTGAG	This study
Δ717wa_F	GCAAGCCAATTTATCCCTGAC	This study
Δ717wa_R	CTTTACATGAATGCTCGTATCGTTATG	This study
YH374A-F	CCGGTATTTTGGCTGATATTGGTAAAGCGC	This study
YH374A-R	CTCTTCTGGCGAGCTTTTTATCC	This study
716-F	AAGCCATGTAATTGAGCATCG	This study
717-R	GAGCCGAGTTTATCGCACC	This study
717-F	GCAAGCCAATTTATCCCTGAC	This study
718-R	TTAGGGCTATCTTGTTGGGTGG	This study
717pe9	TTTATCCCTGACTTTATTTTTTGC	This study
16S-RTF	GGAGTACGGTCGCAAGATTAAA	[4]
16S-RTR	CTAGCGGATTCTCTCAATGTCAA	[4]
RNaseY_RTF	TCTATATCCACCCCGCTGAC	This study
RNaseY_RTR	GTCGTGTGATAGGCAAAGACG	This study
536-RTF	AGGGCGGTGAACTTACAAAG	[4]
536-RTR	CGTTTTTCAGACACCATCAGG	[4]
NikS-RTF	GCGAAAGGCGTATTATATTCCTTCCTTC	This study
NikS-RTR	GGGGAAAGGTAAGAGCGTCAGG	This study
RepG-RTF	CAAGACAAAGGGAAAGGAGGGG	This study
RepG-RTR	CATTCCTTATGGTTTGGTTGGCAC	This study
Flik5-RTF	CCCTAAAAGATTTGCTCAACCAC	This study
FliK5-RTR	CTCCATTAGGCTCTTTTTCATTCTTG	This study
115RTF	ACCGCAGATAAAGCGATGGA	[4]
115RTR	GAGCGCTCTTCGGCTTTCTA	[4]
870RTF	GATGCAAGCCCACCAAATCG	[4]
870RTR	GCGCAGTAGCGATGAGTTTG	[4]

536pe17	AACGATTTGTTTGTTTATGC	[4]
536pe20	TTTGCTAATTTGGTTGTTCC	[4]
5S-F	CGACCTACATTCCCACTCTTG	This study
T7-cncR1-ivtF2	TAATACGACTCACTATAGGGTGTTTTTTAGATGG	This study
cncR1-term1-ivtR	AAAGAAGTAGTTCAGGGCGG	This study
cncR1-term2-ivtR	GATAGAGCTGATATTGAACAGCC	This study
T7-FliK-ivtF2	TAATACGACTCACTATAGGGATGTAAAATAAAAGAGTTG	This study
FliK-ivtR	AAACTTGCTTAAAATCACGC	This study
FliKOPP-ivtF	ATGTAAAATAAAAGAGTTGTTTTAAAGGATAACC	This study
T7-FliKOPP-ivtR	TAATACGACTCACTATAGGGCAAACTTGC	This study
Y_C-FLAG_Fw	ATCATCTTTATAATCTTGCTTGAGCGTAGCGG	This study
Y_C-FLAG_Rev	GATGATAAATAATCAAGCTTTTTTCCCGCACC	This study
YKO-up-F_26695 P12	ATCGCCACAGAATACGCGCATTTG	This study
YKO-up-R-T_26695 P12	CTAGATTTAGATGTCTAAAAAGCTTATCGATCGCTAAAAAGATAGAAGAGAGCGC	This study
YKO-down-F-T_26695 P12	CCTCAAATGGTTCGCTGGGTTTATCGATTAGCTGAAGCGCTTTTTAAAGCGGC	This study
YKO-down-R_P12	TTAGGGCATGAGCTTGACATCAG	This study
VS536F5	GTTTGTTGAAAGAATGTTTGAATTGT	This study
VS536R5	CCTTTGTAAGTTCACCGCCC	This study
VS536F6	ACTGAGAGAAACGAGTAGCAAAAG	This study
VS536R6	TTTCTTATGGGGCAGGGGTG	This study
536RTR_P12	AGGGTATTCTTTGGGATTTTGAGC	This study
536RTF_P12	AAGGCGGTGAACCTATAAAGG	This study
536R5_P12	CCTTTATAGGTTCACCGCCT	This study
536R6_P12	TTTCTTATGGAGCAGGGGTG	This study

Table S4: Top 10 Dali server hits, related to: Bioinformatics analysis of the *H. pylori* RNase Y structure

**Protein**	**PDB**	**Z-score**	**RMSD (Angstrom)**	**Sequence identity (%)**
Polynucleotide phosphorylase, PNPase	6HQ2	11.3	7.0	21
Fuse element of the c-myc oncogene	1J4W	10.8	2.2	23
RNA binding protein, Fubp1	6Y2D	10.4	2.1	26
Archaeal exosome RNA binding protein	2BA0	10.3	7.4	19
Polynucleotide phosphorylase from *Coxiella burnetii*	4NBQ	10.2	22.1	9
Msl5 protein in complex with RNA	4WAL	10.1	6.0	9
C. crescentus PNPase bound to RNA	4AM3	10.0	26.0	15
Polyribonucleotide nucleotidyltransferase	4NBQ	10.0	25.2	10
KH1 domain of human RNA-binding E3 ubiquitin-protein ligase MEX-3C complex with RNA	5WWW	9.9	2.0	17
QUA1-KH domain of T-STAR in complex with UAAU RNA	5ELT	9.9	9.8	13

Table S5: List of up- and down-regulated genes deriving from RNA-sequencing analysis, related to: RNase Y is a non-essential enzyme involved in the regulation of a narrow spectrum of targets

Functional Category	Gene Name	Log2 FC	padj	Common Name	Description
**Down-regulated genes**					
Coenzyme transport and metabolism	HPG27_RS08000	-2.89	2,9E-6	*HPG27_RS08000*	ThiF family adenylyltransferase
Replication, recombination and repair	HPG27_RS07440	-1.19	6,2E-05	*HPG27_RS07440*	Dam family site-specific DNA-(adenine-N6)-methyltransferase
	HPG27_RS04725	-1.04	2,7E-26	*HPG27_RS04725*	dynamin-like GTPase family protein
Lipid transport and metabolism	HPG27_RS04170	-1.08	4,4E-32	*HPG27_RS04170*	phosphatase PAP2 family protein
Unknown or hypothetical	HPG27_RS08695	-2.25	2,7E-11	*HPG27_RS08695*	hypothetical protein
	HPG27_RS08605	-2.09	3,7E-212	*HPG27_RS08605*	50S ribosome-binding GTPase
	HPG27_RS00420	-2.00	6,1E-32	*HPG27_RS00420*	outer membrane protein
	HPG27_RS07995	-1.92	8,8E-08	*HPG27_RS07995*	MFS transporter
	HPG27_RS08600	-1.87	1,1E-19	*HPG27_RS08600*	hypothetical protein
	HPG27_RS00340	-1.63	1,3E-02	*HPG27_RS00340*	SMI1/KNR4 family protein
	HPG27_RS04990	-1.61	2,8E-03	*HPG27_RS04990*	hypothetical protein
	HPG27_RS08660	-1.56	3,0E-04	*HPG27_RS08660*	VirB4 family type IV secretion system protein
	HPG27_RS04730	-1.47	8,5E-34	*HPG27_RS04730*	GTPase
	HPG27_RS03555	-1.45	4,9E-54	*leoA*	50S ribosome-binding GTPase
	HPG27_RS05940	-1.43	5,7E-18	*HPG27_RS05940*	hypothetical protein
	HPG27_RS03940	-1.35	3,6E-04	*fecA2*	TonB-dependent receptor family protein
	HPG27_RS08465	-1.30	4,7E-02	*HPG27_RS08465*	hypothetical protein
	HPG27_RS05805	-1.20	2,8E-50	*hofH*	outer membrane beta-barrel protein
	HPG27_RS01130	-1.17	2,0E-28	*HPG27_RS01130*	sulfite exporter TauE/SafE family protein
	HPG27_RS08050	-1.17	4,9E-31	*HPG27_RS08050*	exonuclease VII large subunit
	HPG27_RS08485	-1.11	5,3E-05	*HPG27_RS08485*	hypothetical protein
	HPG27_RS01135	-1.10	1,5E-34	*hopM*	Hop family outer membrane protein
	HPG27_RS08510	-1.10	1,9E-04	*HPG27_RS08510*	DNA methyltransferase
	HPG27_RS04890	-1.08	6,5E-04	*ptc1*	protein phosphatase 2C domain-containing protein

	HPG27_RS04900	-1.03	4,0E-17	*terY*	VWA domain-containing protein
**Up-regulated genes**					
Coenzyme transport and metabolism	HPG27_RS04130	1,01	1,9E-13	*coaBC*	phosphopantothenoylcysteine decarboxylase/phosphopantoth enate-cysteine ligase
	HPG27_RS03705	1,23	1,2E-128	*HPG27_RS03705*	5-formyltetrahydrofolate cyclo-ligase
	HPG27_RS02940	1,49	1,7E-105	*hemE*	uroporphyrinogen decarboxylase
Posttranslational modification, protein turnover, chaperones	HPG27_RS01325	1,03	9,1E-20	*clpB*	AAA family ATPase
	HPG27_RS00070	1,11	5,4E-17	*groEL*	chaperonin
	HPG27_RS04050	1,21	4,4E-56	*trxB*	thioredoxin-disulfide reductase
	HPG27_RS05325	1,30	6,3E-55	*dsbC*	thioredoxin fold domain-containing protein
	HPG27_RS01900	1,32	9,1E-119	*ftsH*	ATP-dependent zinc metalloprotease
	HPG27_RS01125	1,80	3,4E-59	*msrB*	peptide-methionine (R)-S-oxide reductase
Amino acid transport and metabolism	HPG27_RS00380	1,06	2,0E-04	*ureB*	urease subunit beta
	HPG27_RS02765	1,22	1,5E-55	*pepA*	leucyl aminopeptidase
Amino acid and nucleotide transport and metabolism	HPG27_RS04490	1,51	8,9E-66	*carB*	carbamoyl-phosphate synthase large subunit
Cell motility	HPG27_RS04255	1,07	1,8E-55	*flgE*	flagellar hook protein
	HPG27_RS00600	1,17	1,4E-66	*flaB*	flagellin B
Translation, ribosomal structure and biogenesis	HPG27_RS05880	1,20	5,5E-40	*HPG27_RS05880*	tRNA 2-thiocytidine(32) synthetase TtcA
Amino acid transport and metabolism	HPG27_RS03370	1,24	3,4E-06	*HPG27_RS03370*	hydantoinase B/oxoprolinase family protein
Defense mechanisms	HPG27_RS07830	1,30	6,4E-55	*tsaA*	peroxiredoxin
Host-pathogen interaction, nucleoid	HPG27_RS01220	2,93	3,2E-218	*napA*	neutrophil activating protein
Inorganic ion transport and metabolism	HPG27_RS05900	1,52	9,4E-33	*Cah*	carbonic anhydrase
Replication, recombination and repair	HPG27_RS02120	1,32	1,1E-10	*rarA*	replication-associated recombination protein A
	HPG27_RS01240	1,59	5,3E-40	*deaD*	DEAD/DEAH box helicase

Lipid transport and metabolism	HPG27_RS01890	1,33	1,6E-40	*pssA*	CDP-diacylglycerol--serine O-phosphatidyltransferase
Biogenesis of Cell wall, membrane, envelope	HPG27_RS02945	1,44	8,0E-68	*hefA*	efflux RND transporter outer membrane subunit
	HPG27_RS06905	1,51	4,6E-65	*comL,bamD*	outer membrane protein assembly factor
	HPG27_RS06440	1,57	5,6E-150	*HPG27_RS06440*	glycosyltransferase family 9 protein
	HPG27_RS07880	1,57	2,2E-129	*dniR*	lytic transglycosylase domain-containing protein
Unknown or hypothetical	HPG27_RS04135	1,06	5,5E-21	*HPG27_RS04135*	hypothetical protein
	HPG27_RS05850	1,14	3,5E-72	*hopQ*	Hop family adhesin
	HPG27_RS04430	1,86	3,7E-39	*fliK*	flagellar hook-length control protein
	HPG27_RS03365	1,16	1,4E-05	*HPG27_RS03365*	hydantoinase/oxoprolinase family protein
	HPG27_RS03510	1,18	7,6E-33	*sabA*	Hop family adhesin
	HPG27_RS06645	1,27	2,3E-12	*HPG27_RS06645*	ATP-binding protein
	HPG27_RS04465	1,37	6,7E-18	*alpA*	Hop family adhesin
	HPG27_RS05430	1,39	2,8E-48	*HPG27_RS05430*	hypothetical protein
	HPG27_RS04045	1,45	1,1E-48	*trxA*	thioredoxin
	HPG27_RS01895	1,47	3,6E-23	*HPG27_RS01895*	hypothetical protein
	HPG27_RS07650	1,55	2,3E-25	*horL, omp30*	outer membrane protein
	HPG27_RS01150	1,67	8,0E-165	*hopA*	Hop family outer membrane protein
	cncr1	1,74	6,9E-61	*cncR1*	HsrA regulated ncRNA
	HPG27_RS04495	1,94	1,1E-68	*HPG27_RS04495*	Bax inhibitor-1/YccA family protein
	HPG27_RS07230	2,75	6,0E-141	*trx2*	thioredoxin family protein
	HPG27_RS00305	3,05	3,2E-218	*HPG27_RS00305*	hypothetical protein

REFERENCES

1. Delorenzi, M., and Speed, T. (2002). An HMM model for coiled-coil domains and a comparison with PSSM-based predictions. Bioinformatics *18*. https://doi.org/10.1093/bioinformatics/18.4.617.

2. Lupas, A., Van Dyke, M., and Stock, J. (1991). Predicting coiled coils from protein sequences. Science (1979) *252*. https://doi.org/10.1126/science.252.5009.1162.

3. Zimmermann, L., Stephens, A., Nam, S.Z., Rau, D., Kübler, J., Lozajic, M., Gabler, F., Söding, J., Lupas, A.N., and Alva, V. (2018). A Completely Reimplemented MPI Bioinformatics Toolkit with a New HHpred Server at its Core. J Mol Biol *430*. https://doi.org/10.1016/j.jmb.2017.12.007.

4. Vannini, A., Roncarati, D., and Danielli, A. (2016). The cag-pathogenicity island encoded CncR1 sRNA oppositely modulates Helicobacter pylori motility and adhesion to host cells. Cellular and Molecular Life Sciences *73*. https://doi.org/10.1007/s00018-016-2151-z.
